# Whole genome characterization of thermophilic *Campylobacter* species isolated from dairy manure in small specialty crop farms of Northeast Ohio

**DOI:** 10.3389/fmicb.2023.1074548

**Published:** 2023-03-21

**Authors:** Loic Deblais, Hyein Jang, Mike Kauffman, Jayanthi Gangiredla, Marianne Sawyer, Saritha Basa, Jelmer W. Poelstra, Uma S. Babu, Lisa M. Harrison, Kelli L. Hiett, Kannan V. Balan, Gireesh Rajashekara

**Affiliations:** ^1^Department of Animal Sciences, Center for Food Animal Health, The Ohio Agricultural Research and Development Center, The Ohio State University, Wooster, OH, United States; ^2^Center for Food Safety and Applied Nutrition (CFSAN), Office of Applied Research and Safety Assessment (OARSA), U.S. Food and Drug Administration, Laurel, MD, United States; ^3^Molecular and Cellular Imaging Center, The Ohio Agricultural Research and Development Center, The Ohio State University, Wooster, OH, United States

**Keywords:** *Campylobacter jejuni*, *Campylobacter coli*, whole genome sequencing, dairy manure, small specialty crop farm, resistome, virulome, prophage

## Abstract

**Introduction:**

With more public interest in consuming locally grown produce, small specialty crop farms (SSCF) are a viable and growing segment of the food production chain in the United States.

**Methods:**

The goal of this study was to investigate the genomic diversity of *Campylobacter* isolated from dairy manure (*n* = 69) collected from 10 SSCF in Northeast Ohio between 2018 and 2020.

**Results:**

A total of 56 *C. jejuni* and 13 *C. coli* isolates were sequenced. Multi-locus sequence typing (MLST) identified 22 sequence types (STs), with ST-922 (18%) and ST-61 (13%) predominant in *C. jejuni* and ST-829 (62%) and ST-1068 (38%) predominant in *C. coli*. Interestingly, isolates with similar genomic and gene contents were detected within and between SSCF over time, suggesting that *Campylobacter* could be transmitted between farms and may persist in a given SSCF over time. Virulence-associated genes (*n* = 35) involved in the uptake and utilization of potassium and organic compounds (succinate, gluconate, oxoglutarate, and malate) were detected only in the *C. jejuni* isolates, while 45 genes associated with increased resistance to environmental stresses (capsule production, cell envelope integrity, and iron uptake) were detected only in the *C. coli* isolates. *Campylobacter coli* isolates were also sub-divided into two distinct clusters based on the presence of unique prophages (*n* = 21) or IncQ conjugative plasmid/type-IV secretion system genes (*n* = 15). *Campylobacter coli* isolates harbored genes associated with resistance to streptomycin (*aadE-Cc*; 54%) and quinolone (*gyrA-T86I*; 77%), while *C. jejuni* had resistance genes for kanamycin (*aph3’-IIIa*; 20%). Both species harbored resistance genes associated with β-lactam (especially, *blaOXA-193*; up to 100%) and tetracycline (*tetO*; up to 59%).

**Discussion/Conclusion:**

Our study demonstrated that *Campylobacter* genome plasticity associated with conjugative transfer might provide resistance to certain antimicrobials and viral infections *via* the acquisition of protein-encoding genes involved in mechanisms such as ribosomal protection and capsule modification.

## Introduction

*Campylobacter* is a leading cause of bacterial foodborne gastroenteritis worldwide and is a major public health problem (Moffatt et al., 2021; Mortensen et al., 2021). It has been estimated that 2.4 million people are affected by *Campylobacter* in the United States annually, causing a $1.3 billion deficit in medical care (Zhou et al., 2022). Campylobacteriosis is associated with abdominal pain, fever, and bloody diarrhea, but can also lead to Guillain-Barre syndrome, Miller Fisher syndrome, and reactive arthritis (Kassem et al., 2016; Scallan Walter et al., 2020). Thermophilic *Campylobacter* (especially, *C. jejuni*) are the leading cause of campylobacteriosis cases (90%; Kassem et al., 2016), and can be frequently detected in livestock and livestock-based products (i.e., beef and poultry; Buzby et al., 1997; An et al., 2018; Halpin et al., 2018; Thépault et al., 2018; Scharff, 2020; Heimesaat et al., 2021).

Small specialty crop farms (SSCF) are a viable and growing segment of the food production chain in the United States (valued at $4.7 billion in the Midwest in 2012; Midwest Climate and Specialty Crops, n.d.). With society looking more towards locally grown produce as their source of fresh food, the farmers provide a direct and local connection between producer and consumer. However, these farms frequently practice mixed farming (animal and vegetable farming; Kim, 2016). Further, biological amendments produced by these farms are applied as a natural fertilizer for vegetable production. As a consequence, this practice may increase the risk that crops generated on SSCF are contaminated with foodborne pathogens present in livestock feces (Sharma and Reynnells, 2016; Ramos et al., 2019; Nutrition, 2021). In addition, antibiotics may be used to treat the animals, creating a risk for emergence of potential antimicrobial resistance (AMR) organisms and genes being transferred to the soil when manure is applied. To date, very little is known about the prevalence of foodborne pathogens in this specific agricultural niche and the impact of SSCF agricultural practices on these pathogens in terms of food safety and public health risks. Since 2016, our team has been working with SSCF in Northeast Ohio to understand agricultural practices used by SSCF and assess the impact of biological amendments on public health and food safety in Ohio (Hailu et al., 2021). We demonstrated that manure (dairy and poultry) collected from these farms between 2016 and 2020 harbored thermophilic bacteria such as *Campylobacter* spp. (8%), *Listeria monocytogenes* (7.9%), *Escherichia coli* O157 (1.8%), and *Salmonella* spp. (1.5%; Hailu et al., 2021). The majority of the *Campylobacter* isolates (57.3%) from this study possessed multiple drug resistance genes (especially, *blaOXA-61*, *tetO*, and *aadE*) based on conventional PCR analysis. Thus, our previous study, as well as studies conducted by other groups, have highlighted the potential public health and food safety risks associated with the use of biological amendments in SSCF (Ramos et al., 2019, 2021; Black et al., 2021; Hailu et al., 2021).

This study presents whole genome sequencing (WGS) analyses of *Campylobacter* spp. isolated from diary manure from a longitudinal study performed between 2018 and 2020 in 10 SSCF of Northeast Ohio. A total of 69 thermophilic *Campylobacter* isolates were recovered from the manure samples. Specifically, the goal of this study was to investigate the genomic diversity of *Campylobacter* isolated from dairy manure. We focused on the variable-genome, -resistome, and -virulome to determine how their predicted metabolic capabilities might contribute to their fitness in a specific niche. Correlation analyses between the whole genome sequence and metadata (farm location, sample collection, sequence type, and clonal complex) were performed to better understand the spatiotemporal dynamics of thermophilic *Campylobacter* in SSCF of Northeast Ohio.

## Materials and methods

### Farm selection and sample collection

**Figure 1:**
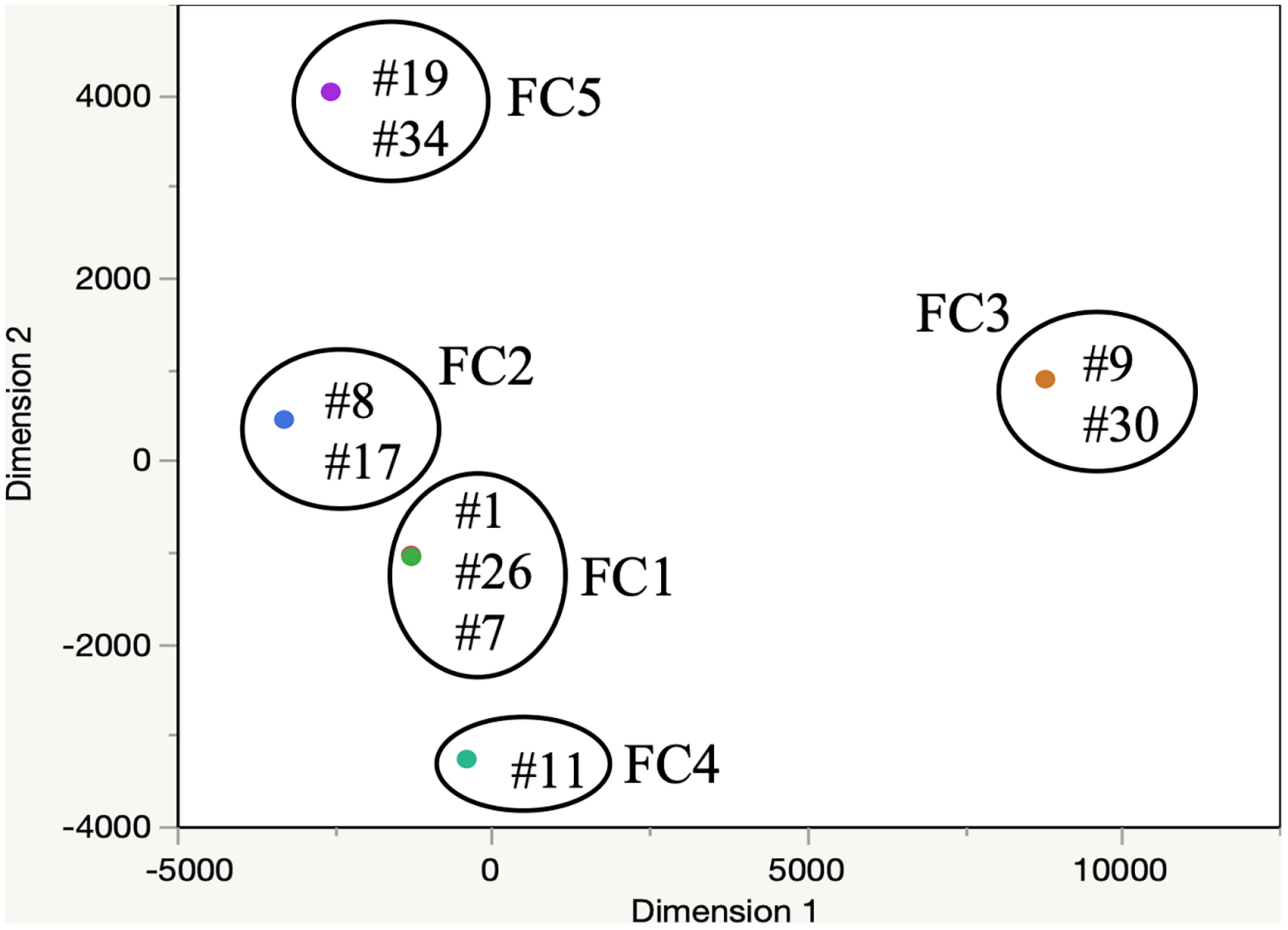
Multi-dimensional scaling of 10 SSCF positive for thermophilic *Campylobacter.* Dimensions 1 and 2 represent distance between farms in meters. Each dot represents a farm selected for the study. #, farm ID; FC, farm cluster.

A total of 10 small specialty crop farms (SSCF) in Northeast Ohio (United States) were selected for this study, based on their availability for longitudinal sampling throughout the study period (2018–2020; Hailu et al., 2021). Farms were asked to continue performing management activities, which followed normal and customary practices of producers utilizing limited mechanization. The spatial distribution of the sample collection sites is provided in Figure 1. Each farm cluster (FC; *n* = 5) was created based on the geographic proximity of the farms with each other. The dairy manure was obtained from open dairy heifers housed on a bedded pack during the winter and raised on pasture in the summer. Dairy manure was not treated by the farmers. Dairy manure samples were collected monthly across the 10 SSCF between 2018 and 2020 (*n* = 140 total samples). Some samples were missing due to events surrounding the COVID-19 pandemic. Samples were collected aseptically into Nasco Whirl-Pak™ bags (Fisher Scientific, Waltham, MA, United States) and stored on ice until processed immediately in the lab.

### Isolation of thermophilic *Campylobacter* from manure

Manure samples (25 g) were resuspended into 225 mL of phosphate buffered saline (PBS; Fisher Scientific, Waltham, MA, United States; 1:10 ratio), homogenized, serial diluted (10-fold), plated on RAPID’ *Campylobacter* (BioRad, Hercules, CA, United States), and then incubated at 42°C in microaerobic conditions (5% O_2_, 10% CO_2_, and 85% N_2_) for 48 h. Resuspended manure samples (1 mL) were also enriched for 48 h at 42°C in 9 mL of Preston enrichment broth containing *Campylobacter* growth supplements (product No. CM067, Lysed Horse Blood product No. SR0048, Preston *Campylobacter* Selective Supplement product No. SR0117 and *Campylobacter* Growth Supplement product No. SR0232; Oxoid Ltd., Cambridge, United Kingdom; 1:10 ratio), plated on RAPID’ *Campylobacter*, and incubated under microaerobic condition at 42°C for 48 h. Colonies growing on RAPID’ *Campylobacter* plates were sub-cultured onto a fresh modified charcoal cefoperazone deoxycholate agar (mCCDA; Fisher Scientific, Waltham, MA, United States) plated and incubated at 42°C for 48 h under microaerobic conditions. A *Campylobacter* genus-specific colony-PCR was performed on all the *Campylobacter*-suspected colonies, as described below. Isolates confirmed to be *Campylobacter* were frozen at −80°C in *Brucella* broth supplemented with 30% glycerol (v/v).

### *Campylobacter* genus-specific colony PCR

Colony-PCR was performed on all isolates suspected to be *Campylobacter* using genus-specific primers (MD16S F: ATCTAATGGCTTAACCATTAAAC; C1228R: GGACGGTAACTAG TTTAGTATT; Final concentration = 0.11 μM; product size = 857 bp; Denis et al., 1999). Briefly, colonies were resuspended in 100 μL sterile DNase-free water and boiled at 95°C for 10 min. The product was centrifuged for 10 min at 4,000 ×*g* and 2 μL of the lysate was used for the colony-PCR (25 μL final volume; 25 cycles). The following program was used: one cycle of 10 min at 95°C, 35 cycles of 30s at 95°C, 1.5 min at 59°C, 1 min at 72°C, and a final extension step of 10 min at 72°C. PCR products were visualized using 2% agarose gels. Isolates confirmed as *Campylobacter* by PCR were selected for whole genome sequencing.

### DNA extraction and whole genome sequencing and assembly

Genomic DNA from pure *Campylobacter* isolates (*n* = 69) was extracted using the Wizard Genomic DNA Purification kit (Promega, Madison, WI, United States). The DNA was quantified using a Qubit dsDNA BR assay kit (Invitrogen, Thermo Fisher Scientific, Waltham, MA, United States) and Qubit 3.0 fluorometer (Invitrogen). The extracted DNA samples were diluted in molecular biology grade nuclease-free deionized water (Thermo Fisher Scientific) to a final concentration of 0.2 ng/μL for whole genome sequencing (WGS). Genomic libraries were constructed using the Nextera XT DNA sample preparation kit as described in the manufacturer’s protocol (Illumina, San Diego, CA, United States) and sequenced on the MiSeq platform using 500 cycles of paired-end reads (Illumina). Quality control of the FASTQ datasets (raw reads) obtained from each sequence run was assessed using FastQC (Andrews, 2010). Low-quality reads < Q20 were trimmed and adaptor sequences were removed using the default parameter in Trimmomatic (Bolger et al., 2014). Trimmomatic modifications were conducted with a sliding window trimming option where the number of bases to average across was set to 4 and the average quality required was set to 20. The cleaned reads were subsequently *de novo* assembled using SKESA (Strategic K-mer Extension for Scrupulous Assemblies; Souvorov et al., 2018). Assembly qualities were also evaluated by QUAST (Quality Assessment Tool; Gurevich et al., 2013; Mikheenko et al., 2018). The analytical tools for FastQC, Trimmomatic, SKESA, and QUAST are available on the GalaxyTrakr platform (https://galaxytrakr.org, accessed 27 May 2022) which is an open-source bioinformatics platform maintained by the Center for Food Safety and Applied Nutrition (CFSAN) at the U.S. Food and Drug Administration (Gangiredla et al., 2021).

### Multi-locus sequence typing: Traditional and core genome MLST

Sequence types (STs) of *Campylobacter* genomes investigated in this study were initially analyzed using the traditional seven-loci Multi-locus sequence typing (MLST) scheme based on the following housekeeping genes (*aspA*, *glnA*, *gltA*, *glyA*, *pgm*, *tkt*, and *uncA*; Dingle et al., 2001). Briefly, the genome assemblies were scanned against the PubMLST schemes where the ST of each genome was assigned by comparing the alleles of the seven genes in the MLST open database (Jolley and Maiden, 2010; Seemann, 2022b).

To cover the expanded resolution of the MLST concept, a core genome MLST (cgMLST) profiling against the PubMLST database (Jolley et al., 2018) was performed using a modified Seemann’s MLST tool which used contig files to scan against the Oxford *C. jejuni* and *C. coli* cgMLST scheme (Mulder et al., 2020). The cgMLST scheme describes the genetic variation among the strains by utilizing the allele sequences of 1,343 loci which were defined from genome sequences of 2,472 representative United Kingdom campylobacteriosis isolates including *C. jejuni* and *C. coli* (Cody et al., 2017). The traditional MLST tool was used on the GalaxyTrakr platform (accessed 28 April 2022) while the cgMLST tool was accessed (28 April and 16 December 2022) from an open-source platform^1^ to query the genomes against the current *C. jejuni* and *C. coli* scheme. The closest ST match for these isolates were assigned using the cgMLST scheme on the PubMLST *Campylobacter jejuni/coli* typing database^2^ (Cody et al., 2017; Jolley et al., 2018).

### Taxonomic classification and annotation

To identify the isolates at the genus and species level, the sequence data were analyzed by Kraken 2, which is a *k*-mer-based taxonomic sequence classifier using *k*-mers of 35 bp (Wood and Salzberg, 2014; Wood et al., 2019). The Kraken 2 algorithm assigns taxonomic labels by matching each *k*-mer within a query sequence to the lowest common ancestor (LCA) of the pre-computed database of genomes containing the given *k*-mer (Wood et al., 2019). In addition, the genomes were annotated by uploading the assemblies of FASTA datasets through the National Center for Biotechnology Information (NCBI) prokaryotic genome annotation pipeline (PGAP) with its best-placed reference protein set GeneMarkS+ application (Haft et al., 2018; Li et al., 2021). Assembled genomes were also annotated using RAST (Aziz et al., 2008; Overbeek et al., 2014) to provide an overall picture of the gene content across the *Campylobacter* isolates.

### Identification of antimicrobial resistance and virulence genes based on WGS

The predicted antimicrobial resistance (AMR) genes were identified using the National Antimicrobial Resistance Monitoring System (NARMS) *Campylobacter* workflow on GalaxyTrakr which uses BLAST techniques against the NCBI’s comprehensive AMR gene database (Feldgarden et al., 2019a,b, 2021). The AMRFinderPlus tool accessed from the GalaxyTrakr platform was used to detect acquired AMR genes in bacterial proteins or assembled nucleotide sequences, along with point mutations that were cross-referenced with a core set of AMR elements and the expanded subset of reference genes related to biocide, stress response, and virulence factors (Feldgarden et al., 2021). The AMRFinderPlus tool was designed to utilize the Reference Gene Catalog database of NCBI Pathogen Detection, which consists of 6,428 genes (5,588 AMR genes, 210 stress response genes, and 630 virulence genes), 627 hidden Mark models (HMMs), and 682 point-mutations (Feldgarden et al., 2021). Virulence gene profiles of *Campylobacter* isolates were determined by BLASTN comparison against the virulence factor database (VFDB) with the threshold of 80% identity and 80% sequence coverage (Chen et al., 2016; Liu et al., 2019) by using an ABRicate tool which performed mass screening of contigs for antimicrobial resistance and virulence genes (Seemann, 2022a).

### Phylogenetic analysis based on single nucleotide polymorphism

To understand relatedness among the strains, the *Campylobacter* genome assemblies were analyzed using the CFSAN SNP Pipeline (Davis et al., 2015) which is accessible on the GalaxyTrakr platform. The single nucleotide polymorphism (SNP) pipeline performs the alignment of mapped reads of sequences to a reference genome, which creates high-quality SNP matrices for sequences and determines phylogenetic relatedness among the strains (Davis et al., 2015). The SNP analysis was conducted against the reference genome chosen by the SNP pipeline, and a reference strain was chosen based on the best assembly metrics from the quast report, such as the one with the longest N50. For this analysis, sample #112962 (Biosample accession # SAMN29955749) was selected as a reference to build the tree. The phylogenetic tree was constructed using the Neighbor-Joining method (Saitou and Nei, 1987). The evolutionary analyses were employed using MEGA7 software (Kumar et al., 2016, p. 7), with evolutionary distances computed using the Maximum Composite Likelihood method (Tamura et al., 2004).

### Plasmid annotation

Assembled genomes were scanned for plasmids using PlasmidFinder v. 2.1.6 (Carattoli et al., 2014), which detects plasmids *via* replicons only, and MOB-Suite v. 3.1.0 (Robertson and Nash, 2018), which detects plasmids with a database containing replicons, relaxases, and complete plasmid sequences. PlasmidFinder was run using the plasmidfinder.py script, after downloading the database with the download-db.sh script (on 16 July 2022), with a minimum coverage threshold of 0.60 (the default) and an identity threshold of 0.95 (the default for the webserver, whereas the default for the Python script is 0.90). MOB-Suite was run *via* the “mob_recon” command, after running “mob_init” to download the databases (on 18 November 2022) with default parameters.

### Statistical analyses

Genomic data were synchronized with the metadata (e.g., collection time point and location, and source) and analyzed using JMP Pro 16 software (SAS Institute Inc., Cary, NC, United States). The analyses described below focus essentially on the variable genomes (protein-encoded genes not consistently detected within all the *C. jejuni* or *C. coli* genomes) and not the core genome (protein-encoded genes consistently detected within all the *C. jejuni* or *C. coli* genomes). Multi-dimensional scaling analysis combined with K-means clustering were used to create a two-dimensional plot displaying the farm geographic distribution and associated clusters based on the GPS coordinates of the SSCF. Hierarchical clustering analysis was conducted to identify similar variable gene profiles among the 69 *C. jejuni* and *C. coli* isolates. Principal component (PCA) and agglomerative hierarchical clustering (HCA) analyses were used to study the distribution of the *Campylobacter* isolates based on their functional genomic profiles and cgMLST. Multi-correspondence analysis (MCA) combined with a Chi^2^ test were used to determine whether the prevalence of certain genes was influenced by the *Campylobacter* species, SSCF, and sample collection time point (e.g., year and month of collection). MCA and multivariate analyses were used to identify co-occurrence of specific genes for a given *Campylobacter* species. In addition, an association study (also called produce market analysis) was conducted to determine whether the prevalence of specific genes could be linked with specific geographic or temporal parameters associated with the isolates. A minimal confidence level of 95% and lift of 2 were used to identify associations of statistical importance.

## Results

### Thermophilic *Campylobacter* are frequently detected in dairy manure from Northeast Ohio

Out of the 140 dairy manure samples collected from the 10 SSCF between 2018 and 2020, 49% (*n* = 69) were positive for thermophilic *Campylobacter* (Table 1). Dairy manure collected in 2020 and 2019 harbored the highest prevalence (88% and 69%, respectively) compared to 2018 (18%; *p* < 0.001). On the other hand, dairy manure collected in 2020 harbored the lowest abundance of *Campylobacter* (2.92 [IC95%: 2.61–3.22]) log CFU/g compared to 2019 and 2018 (3.89 [IC95%: 3.64–4.13] and 3.28 [IC95%: 2.99–3.57] log CFU/g, respectively Table 1; *p* < 0.008). Equivalent *Campylobacter* prevalence (60%–100%) and load (2.76 [IC95%: 2.31–3.21] to 4.21 [IC95%: 3.82–4.60] log CFU/g) in the dairy manure were detected between farms and seasons (*p* > 0.01).

**Table 1:** Prevalence of thermophilic *Campylobacter* spp. in dairy manure from SSCF of Northeast Ohio.

Years	Total number of samples tested	*Campylobacter* prevalence (% and number of samples)		*Campylobacter* load in positive samples (Log_10_ mean [IC 95%])
		Negative	Positive	
2018	65	82% (53)	18% (12)	3.28 [IC95%: 2.99–3.57]
2019	49	31% (15)	69% (34)	3.89 [IC95%: 3.64–4.13]
2020	26	12% (3)	88% (23)	2.92 [IC95%: 2.61–3.22]

### Whole genome sequence of *Campylobacter* isolates

The whole genome analyses revealed 56 *C. jejuni* (genome length = 1,683,112 bp [IC95%: 1,664,247–1,701,977]) and 13 *C. coli* (genome length = 1,691,610 bp [IC95%: 1,680,103-1,703,117]) isolates (Supplementary Table 1). All associated metadata and genome quality statistics are included in Supplementary Table 2. *Campylobacter jejuni* was recovered across all 3 years of sampling, predominantly in 2019 (*n* = 33/56; Table 2). Most of the *C. coli* isolates were recovered in 2020 (*n* = 12/13; Table 2). Isolates were collected in the winter, spring, and summer seasons (*n* = 16–19 *C. jejuni* and 3–6 *C. coli* isolates per season), but rarely in the fall (*n* = 3/56 *C. jejuni* isolates and no *C. coli* isolates).

**Table 2:** Distribution of the sequenced thermophilic *Campylobacter* isolates based on farm ID, farm clusters and years of collection.

Year of collection	Species	Number of isolates	Farm cluster and farm ID									
			Cluster 5		Cluster 2		Cluster 4	Cluster 1			Cluster 3	
			ID#19	ID#34	ID#8	ID#17	ID#11	ID#1	ID#7	ID#26	ID#9	ID#30
2018	*C. coli*	0										
	*C. jejuni*	12	2			2	2	4	1		1	
2019	*C. coli*	1			1							
	*C. jejuni*	33				7	2		4	11	1	8
2020	*C. coli*	12		3	3				6			
	*C. jejuni*	11			8				2		1	
Total		69	2	3	12	9	4	4	13	11	3	8

Empty cells: no *Campylobacter* isolated.

### MLST analysis of the *Campylobacter* isolates

A total of 20 STs were identified among the 56 *C. jejuni* isolates, with ST-922 (*n* = 10) and ST-61 (*n* = 7) being the most predominant (Table 3). These 20 STs belonged to 10 clonal complexes (CCs), with CC-21 (*n* = 26) being the most predominant. The geographic location of the farms affected the type of ST and CC detected. The majority of the ST-61 (*n* = 6/7) were detected in farm #30 in 2019; ST-922 (*n* = 7/10) were detected in farm #26 in 2019 and ST-829 (*n* = 5/8) were detected in farm #7 in 2020. The spatiotemporal distribution of the CC and ST was studied across the 10 farms over 3 years. Similar CCs were detected in FC #1 (farm #1, 7 and 26) and FC #2 (farm #8 and 1 7; Supplementary Figure 1A), which is in concordance with the geographic proximity of both FC (Figure 1). However, FC #3, #4, and #5 harbored different CCs compared to FC #1 and #2 (Supplementary Figure 1A), which is also in agreement with the distant geographic location of the FCs between each other (Figure 1). Two STs (ST-829 [*n* = 8] and ST-1068 [*n* = 5]) were detected among the 13 *C. coli* isolates and belonged to CC-828. The number of STs and CCs (*C. jejuni* and *C. coli* combined) were equivalent across collection years (*n* = 9–12 and 5–7, respectively), but each year a different ST/CC profile was observed (Supplementary Figure 1B). No differences in ST and CC profiles were detected between seasons.

**Table 3:** Multi-locus sequence typing of thermophilic *Campylobacter* isolates using the traditional seven gene schema.

Species	Number of isolates	Farm ID	Farm cluster	Year	*aspA*	*glnA*	*gltA*	*glyA*	*pgm*	*tkt*	*uncA*	ST	CC
*Campylobacter coli*	8	7,8,34	1,2,5	2020	33	39	30	82	113	43	17	829	828
	5	7,8,34	1,2,5	2019, 2020	33	39	30	78	104	43	17	1,068	828
*Campylobacter jejuni*	5	8,17	2	2018, 2020	2	1	1	3	2	1	6	8	21
	2	19,30	3,5	2018, 2019	2	1	1	3	2	1	5	21	21
	2	7,11	1,4	2018, 2019	1	2	3	4	5	9	3	42	42
	3	7,26,30	1,3	2019	4	7	10	4	1	7	1	45	45
	1	1	1	2018	1	2	3	9	5	9	3	11,771	42
	1	8	2	2020	2	1	3	3	5	9	3	11,712	42
	1	19	5	2018	2	4	1	2	7	1	5	48	48
	3	11,26	1,4	2019	9	25	2	10	22	3	6	52	52
	7	7,30	1,3	2019	1	4	2	2	6	3	17	61	61
	1	1	1	2018	4	7	40	4	42	51	1	267	283
	2	8,9	2,3	2019, 2020	10	27	16	19	9	5	9	403	403
	4	1,17	1,2	2018, 2019	1	2	3	3	5	9	3	459	42
	1	9	3	2020	24	2	2	2	10	3	1	464	464
	1	26	1	2019	1	2	3	27	5	9	3	604	42
	4	7,9,17	1,2,3	2018, 2019	2	1	1	3	140	3	5	806	21
	10	1,7,11,26	1,4	2018, 2019	1	1	2	83	2	3	6	922	21
	3	8	2	2020	9	2	4	62	4	5	17	929	21
	2	17,26	1,2	2019	2	1	2	3	2	1	5	982	21
	1	17	2	2019	1	1	2	2	225	3	17	1,244	61
	2	7	1	2020	2	2	4	62	4	5	17	5,261	257

### Potential associations between the spatial and temporal distributions of the *Campylobacter* isolates and their genomic profile

**Figure 2:**
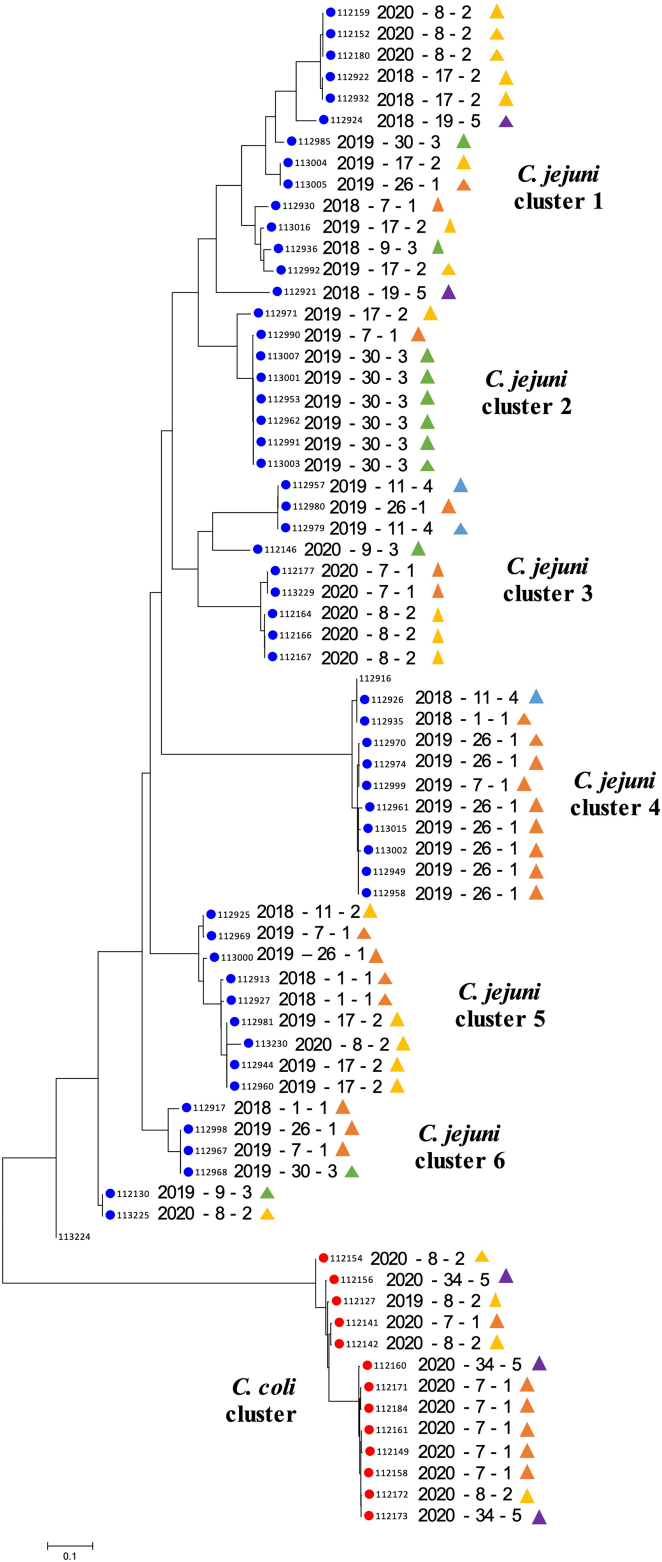
Phylogenetic diversity based on single nucleotide polymorphisms (SNPs) analysis. The phylogenetic tree was constructed using the Neighbor-Joining method. The optimal tree with the sum of branch length = 2.14 is shown. The tree was generated to scale with branch lengths in the same units as those of the evolutionary distances used to infer the phylogenetic tree. The analysis involved 69 genomes. There was a total of 2,748 positions in the final dataset. The scale bar represents a 0.1 base substitution per site. Each row indicates a specific *Campylobacter* isolate (year of collection—farm ID—farm cluster). Blue and red circles indicate *Campylobacter jejuni* and *Campylobacter coli,* respectively. Colored triangles are associated with the farm cluster number; orange, yellow, green, blue and purple triangles indicate farm clusters 1 to 5, respectively (Figure 1).

Distinct SNP signatures were detected between different *Campylobacter* species, as well as, between isolates of the same species (Figure 2). As expected, *C. jejuni* isolates segregated from the *C. coli* isolates. A total of six major *C. jejuni* clusters were observed based on the SNP profiles. The composition of these clusters was associated to some extent with the geographic location, and less with the time of collection (Figure 2). Most of the *C. jejuni* isolates collected from farm #30 (*n* = 6/8) clustered together and displayed identical SNP profiles (cluster 2). The majority of *C. jejuni* isolates collected from FC #1 (*n* = 16/21; farm #1, #7, and #26; Figure 1) clustered away from the other *C. jejuni* isolates (*n* = 31; cluster 4; Figure 2). Interestingly, FC #1 was mainly composed of *C. jejuni* isolated between the summers of 2018 and 2019, and harbored high SNP profile similarity with a *C. jejuni* isolated in early 2018 from farm #11. Farm #11 belongs to FC #4 which is in close proximity to FC #1 (Figure 1). High SNP profile similarities were also observed between the *C. jejuni* isolates collected between FC #1 and FC #2 (farm #8 and #17; cluster 3 and 5; Figure 2). A similar trend was observed with *C. coli* isolates between FC#1, FC#2 and FC#5 (*C. coli* cluster; Figure 2).

The 69 *Campylobacter* isolates were also analyzed using core genome MLST (cgMLST). A total of 869/1,343 alleles were detected across the 69 isolates (Supplementary Figure 2). Overall, a distinct separation between *C. jejuni* and *C. coli* was observed. For the *C. coli* isolates, ST-829 isolates (*n* = 8) clustered away from the ST-1068 isolates (*n* = 5). Interestingly, *C. jejuni* CC-21 isolates were subdivided into four clusters; all *C. jejuni* ST-922 CC-21 isolates (*n* = 10) clustered away from the second (i.e., *C. jejuni* CC-21 ST-8 [*n* = 5]), third (i.e., *C. jejuni* CC-21 ST-929 [*n* = 3]) and fourth cluster (i.e., *C. jejuni* CC-21 ST-806 [*n* = 4] and ST-21 [*n* = 1]). The allelic profile of *C. jejuni* ST-922 CC-21 isolates was also closely similar to *C. jejuni* CC-403, CC-463 and CC-52. Similarly, other *C. jejuni* CC-21 (i.e., ST-806 and ST-982) were closely related to *C. jejuni* CC-48 and CC-61. *Campylobacter jejuni* ST-5261 CC-257 were closely related to of *C. jejuni* ST-929 CC-21 cluster, which itself displayed similarities with *C. jejuni* CC-42 isolates. Only *C. jejuni* ST-8 CC-21 and ST-45 CC-45 isolates stood apart from other *C. jejuni* isolates.

### *Campylobacter jejuni* and *Campylobacter coli* isolates possess distinct gene content profiles

After annotating the genomes using RAST (general gene content profile), VFDB (virulome annotation) and NDARO (resistome annotation), a total of 1,784 annotated genes were detected across the 69 *Campylobacter* isolates. Among them, 35.7% (n = 637/1,784) of annotated genes were not always detected in the 69 *Campylobacter* isolates studied (referred to as variable genes). As observed with the SNP analysis (Figure 2), *C. coli* isolates displayed a different gene content profile compared to the *C. jejuni* isolates. *Campylobacter jejuni* isolates possessed several unique virulence-associated genes involved in the uptake of potassium, carbon utilization (e.g., succinate, gluconate, oxoglutarate and malate), and chemotaxis (*n* = 35; Supplementary Table 3). Similarly, *C. coli* isolates also had unique virulence genes associated with capsule production, cell envelope integrity, and membrane transporters, increasing resistance to environmental stresses (Supplementary Table 3). *Campylobacter coli* isolates also possessed unique sets of genes coding for enzymes (e.g., histidine kinase, acylamide amidohydrolase, methylcitrate dehydratase and synthase, adenine-specific DNA methyltransferase, adenylylsulfate kinase, L-carnitine dehydratase, and methionine synthase), transporters (e.g., *creD*, *yihN*, and *yihY*) essential for the utilization of specific energy sources, and proteins involved in iron availability and/or acquisition (e.g., *ycsG*, hemerythrin, hemolysin, and peroxide stress regulator/ferric uptake regulation protein; Supplementary Table 3).

### Functional genomic analyses of *Campylobacter jejuni* demonstrated associations between the geographic location and sequence type of the isolates

**Figure 3:**
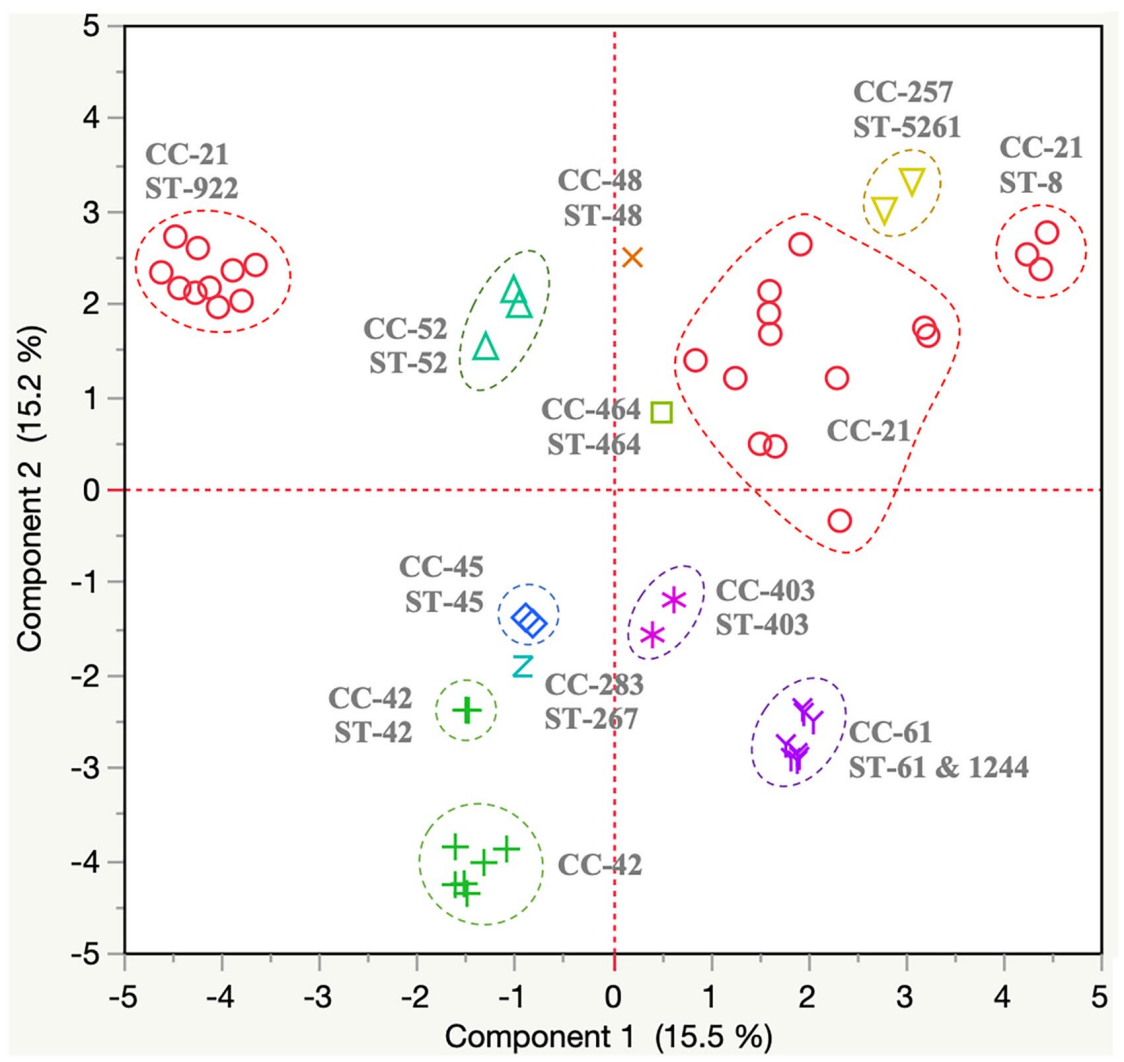
Variable genome distribution of *Campylobacter jejuni* (*n* = 56) isolated from dairy manure in small specialty crops farms in Northeast Ohio between 2018 and 2020. A total of 401 protein-encoding genes were identified as part of the *C. jejuni* variable genome and were used to create this distance matrix plot. Dotted circles indicate specific clonal complex (CC)-sequence type (ST) clusters. The color of each dot within the plot represents a different CC. The principal component plot is defined by component 1 and 2, which explains 15.5% and 15.2%, respectively of variations based on the variable-genome (*n* = 401 protein encoding genes) of the 56 *C. jejuni* isolates.

A total of 401 protein-encoding genes were identified as part of the *C. jejuni* variable genome (all variable genes identified across the 56 *C. jejuni* genomes). Specific gene content profiles (*n* = 12 major clusters; Table 4; Supplementary Table 4) were identified. We observed that the gene content profiles were closely associated with ST of the isolates (Figure 3). Additional details about the variable gene profile distribution observed for each *C. jejuni* isolate are presented in Supplementary Figure 3A.

**Table 4:** Variable genome clusters in *Campylobacter jejuni* isolates.

Cluster ID	Number of isolates	Variable genome associated functions^*^	Clonal complex (CC) and sequence type (ST)
Cj1	51	Lipooligosaccharide synthesis	All but CC-21 ST-929 and CC-257 ST-5261
		O-methyl phosphoramidate capsule modification	
		Methionine metabolism	
Cj2	5	Fatty acid synthesis	CC-21 ST-929 and CC-257 ST-5261
		dmsABC sulfoxide reductase	
		Capsular polysaccharide biosynthesis	
Cj3	52	Pantothenate and rhamnose synthesis	All but CC-45 ST-45 and CC-283 ST-267
		Capsular polysaccharide biosynthesis	
		Fucose utilization	
		Bacteriocin resistance	
Cj4	4	Arsenate resistance	CC-45 ST-45 and CC-283 ST-267
		Glycosaminoglycan biosynthesis	
Cj5	38	Cell surface glycoconjugates (glutamate and legionaminic acid)	All but CC-42
Cj6	22	Fucose utilization	All but CC-42, CC-45 & CC-61
		Glucuronate synthesis	
Cj7	10	Rhamnose, methionine and purine metabolism	CC-21 ST-922
Cj8	7	Type IV secretion system	CC-21 (ST-21, ST922, ST-806) and CC-42 (ST-459, ST-11771)
		IncQ plasmid conjugation transfer	
		cag12 pathogenicity island protein	
Cj9	6	Type IV secretion system	CC-21 (ST-806) and CC-42 (ST-459, ST-11771)
		IncQ plasmid conjugation transfer	
		IncF plasmid conjugation transfer	
Cj10	5	Type IV secretion system	CC-21 (ST-806) and CC-42 (ST-459, ST-11771)
		IncF plasmid conjugation transfer	
		pVir protein	
		Other virulence genes	
Cj11	12	Phage DNA	CC-21 (ST-806, ST-982) and CC-257 ST-5261
		Type IV secretion system	
		Trb conjugation transfer	
Cj12	2	Type VI secretion system	CC-464 ST-464 and CC-403 ST-403

* Additional details about the protein-encoding genes identified in each cluster are described in Supplementary Table 4.

Most *C. jejuni* isolates (*n* = 51/56 isolates) possessed genes associated with lipooligosaccharide synthesis, O-methyl phosphoramidate capsule modification and methionine metabolism (*n* = 12 genes; Cluster Cj1 in Table 4; Supplementary Table 4). On the other hand, five isolates missing the genes from cluster Cj1 possessed genes involved in fatty acid synthesis, *dmsABC* sulfoxide reductase and capsular polysaccharide biosynthesis (*n* = 8 genes; Cluster Cj2 in Table 4; Supplementary Table 4). Additionally, most isolates (*n* = 52/56 isolates) possessed genes associated with pantothenate and rhamnose synthesis, capsular polysaccharide biosynthesis, fucose utilization, and bacteriocin resistance (*n* = 12; Cluster Cj3 in Table 4; Supplementary Table 4). However, the four isolates missing the genes from cluster Cj3 possessed other genes involved in arsenate resistance and glycosaminoglycan biosynthesis (*n* = 5 genes; Cluster Cj4 in Table 4; Supplementary Table 4).

A fraction of the isolates (*n* = 38/56 isolates) possessed genes associated with cell surface glycoconjugates (glutamate and legionaminic acid; *n* = 8 genes; Cluster Cj5 in Table 4; Supplementary Table 4). Among these isolates, 22 *C. jejuni* (especially from FC #2) also harbored genes for the utilization of fucose and glucuronate synthesis (*n* = 6 genes; Cluster Cj6 in Table 4; Supplementary Table 4).

Several isolates from FC #1 (*n* = 10/22 isolates; especially ST-922 CC-21 from farm #26) harbored genes associated with rhamnose, methionine, and purine metabolism (*n* = 10 genes; Cluster Cj7 in Table 4; Supplementary Table 4). The majority of these isolates also possessed type IV secretion systems (T4SS) protein-encoding genes (*virB2,3,5–9,* and *virD4*; *n* = 10 genes), IncQ plasmid conjugative transfer-related genes (*traC, traE, traG and traR*; *n* = 4 genes), and the gene encoding cag pathogenicity island protein (*cag12*; Cluster Cj8 in Table 4; Supplementary Table 4). A similar T4SS/conjugation profile was observed in *C. jejuni* isolates from FC #2 (*n* = 6/17 isolates, especially farm #17). These isolates also harbored genes associated with IncF plasmid conjugative transfer pilus assembly (*n* = 11 genes; *traB,C,E,H,K,L,N,T,U,V,* and *W*; Cluster Cj9 in Table 4; Supplementary Table 4). Further, 5 of these isolates also possessed additional protein-encoding genes associated with T4SS (*virB4,7*) and conjugative transfer (*traG*), hypothetical pVir protein (*n* = 10 genes, pVir0004,7,8,9,12,15,19,20,29,42), and other virulence-associated genes (*n* = 15; Cluster Cj10 in Table 4; Supplementary Table 4).

Another group of isolates (*n* = 12 isolates) harbored genes associated with conjugative transfer (*n* = 6; *trbB,D,E,F,I,L*) and T4SS (virB1), as well as a high abundance of bacteriophage DNA (*n* = 29 genes) especially from the *Escherichia coli* phage protein families Gp (phage lambda) and Mup (phage Mu; Cluster Cj11 in Table 4; Supplementary Table 4). Three isolates harbored type VI secretion system genes (T6SS; *n* = 6 genes; *hcp*, *impB,C,G,I,K*; Cluster Cj12 in Table 4; Supplementary Table 4).

Eighty-one protein encoding genes belonging to the variable genome were not clustered based on the CC or ST of the *C. jejuni* isolates. A majority of the protein-encoding genes (*n* = 27) were labeled as possible, putative, uncharacterized or hypothetical proteins (Supplementary Table 4).

### Functional genomic profile analysis of *Campylobacter coli* demonstrated associations between the geographic location and sequence type of the isolates

A total of 102 protein-encoding genes were identified as part of the *C. coli* variable genome. Specific gene content profiles (*n* = 2 major clusters) were identified based on the ST associated with the isolates (Table 5; Supplementary Table 5). Additional details about the variable gene profile distribution observed for each *C. coli* isolate are presented in Supplementary Figure 3B. Interestingly, the major difference between these two clusters is the presence/absence of genes involved in the release and uptake of DNA and protein. All *C. coli* ST-829 CC-828 (*n* = 8 isolates) possessed phage DNA (*n* = 24 genes, especially from Gp and Mup families) and genes encoding enzymes associated with hexose utilization (glucose, abequose, fucose, mannose, glycerate, glucoronate and rhamnose; *n* = 14 genes; cluster Cc1 in Table 5; Supplementary Table 5). On the other hand, *C. coli* ST-1068 CC-828 (*n* = 5 isolates) possessed genes associated with conjugation transfer (*n* = 4 genes; *traC,E,G*, and *Q*), T4SS (*n* = 10 genes; *virB2,4–11* and *virD4*), capsule modification (*n* = 5 genes), and cell wall synthesis (*n* = 2 genes; cluster Cc2 in Table 5; Supplementary Table 5).

**Table 5:** Variable genome clusters in *Campylobacter coli* isolates.

Cluster ID	Number of isolates	Variable genome associated functions^*^	Clonal complex (CC) and sequence type (ST)
Cc1	8	Phage DNA (Gp and Mup family)	CC-828 ST-829
		Hexose utilization (glucose, abequose, fucose, mannose, glycerate, glucoronate and rhamnose)	
Cc2	5	IncQ plasmid conjugative transfer	CC-828 ST-1068
		O-methyl phosphoramidate capsule modification	
		Cell wall synthesis	

* Additional details about the protein-encoding genes identified in each cluster are described in Supplementary Table 5.

### Antimicrobial resistance profile of *Campylobacter jejuni* and *Campylobacter coli* isolates

**Figure 4:**
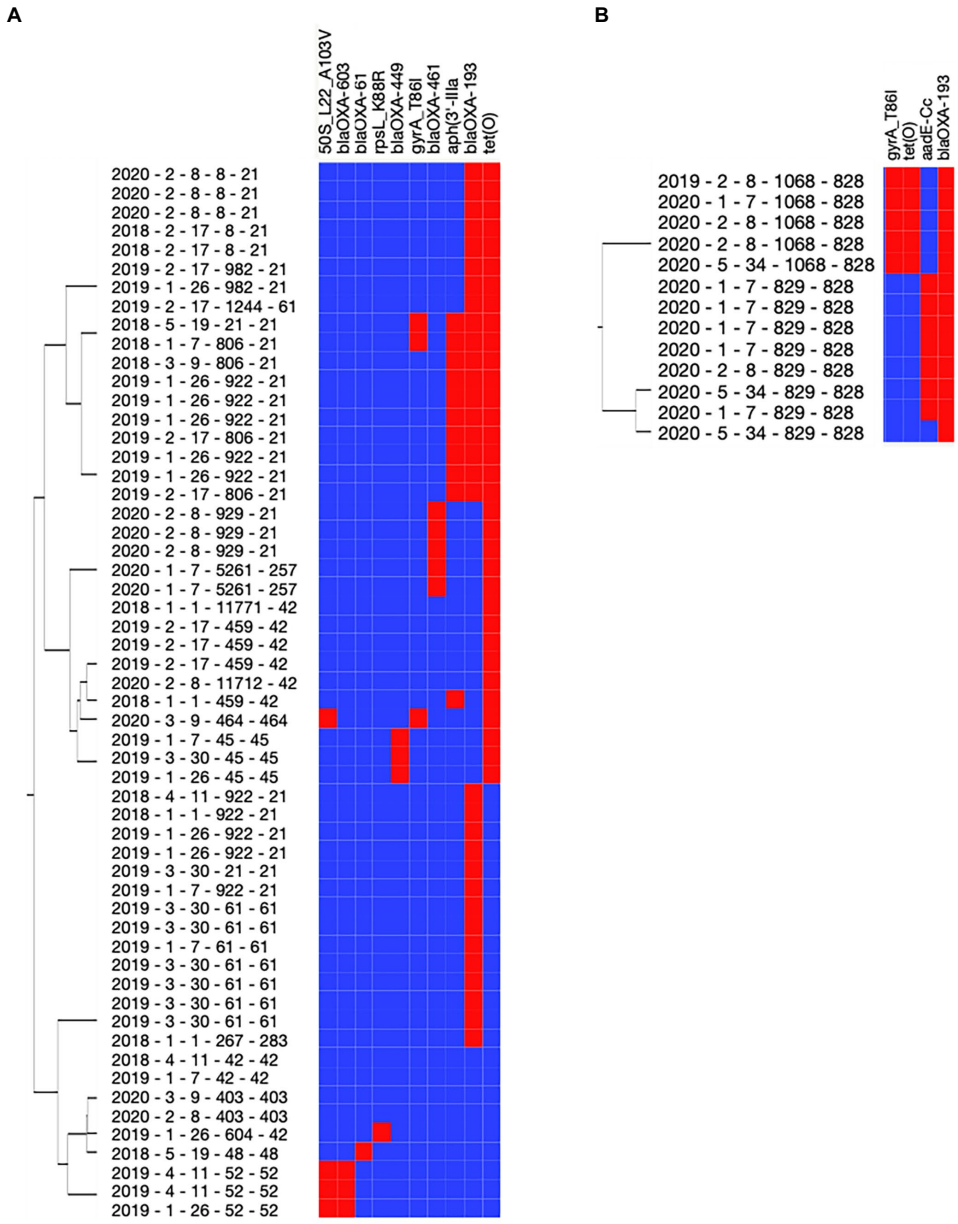
Antimicrobial resistance gene content of *Campylobacter* spp. isolated from dairy manure in small specialty crops farms in Northeast Ohio from 2018 to 2020. Resistome of *Campylobacter jejuni* **(A)** and *Campylobacter coli* **(B)**. Eleven genes were detected across the 69 *Campylobacter* isolates using the NARMS database. The tree associated with the Y-axis displays the similarities of resistome profile among the isolates. Red and blue cells represent genes detected or missing (Y-axis) in the designated *Campylobacter* isolate (X-axis), respectively. Metadata associated with the *Campylobacter* spp. isolates are presented in the following order: year of collection (2018–2020), farm cluster (1–5), farm ID (#1–#34), sequence type (ST), and clonal complex (CC).

Using the National Antimicrobial Resistance Monitoring System for Enteric Bacteria (NARMS) database, a total of 11 antimicrobial resistance genes (ARGs) were detected across the 69 *Campylobacter* isolates (Table 6; Figure 4). Three ARGs (*blaOXA-193, gyrA_T86I,* and *tetO*) were detected in both *C. jejuni* and *C. coli* isolates (Table 6). *Campylobacter jejuni* isolates were characterized with beta-lactamase (*blaOXA-449, blaOXA-461, blaOXA-603*, and *blaOXA-61*), macrolide (*50S_L22_A103V*), and aminoglycoside ARGs (*rpsL_K88R* and *aph3’IIIa*; Figure 4A), while *C. coli* isolates were characterized with an aminoglycoside ARG (*aadE-Cc*; Figure 4B). Interestingly, all *C. coli* isolates also harbored genes encoding proteins associated with resistance to arsenic (arsenate reductase [EC 1.20.4.4] thioredoxin-coupled and arsenical-resistance protein ACR3). All isolates with *aadE-Cc* also harbored *tetO* and *gyrA_T86I* (r^2^ = −0.85; *p* = 0.0002), and all isolates with *tetO* also had *gyrA_T86I* (r^2^ = 1; *p* < 0.0001) in *C. jejuni*. No ARG co-occurrence was detected in *C. coli.* A total of 14 ARG profiles were observed across the 69 isolates (Supplementary Table 6). Most of the isolates possessed genes conferring resistance to at least two antibiotics (*n* = 43/69 isolates). The co-occurrence of genes involved in the resistance to streptomycin (*aadE-Cc*) and beta-lactam (*blaOXA-193*) or to tetracycline (*tetO*), quinolone (*gyrA_T86I*), and beta-lactam (*blaOXA-193*) were predominant in *C. coli* (*n* = 7/13 and 5/13 isolates, respectively; Supplementary Table 7). Similarly, for most *C. jejuni,* the resistance to tetracycline (*tetO*) and beta-lactam (*blaOXA-193, −461 or −449; n* = 16/56 isolates), or to kanamycin (*aph[3’IIIa]*), beta-lactam (*blaOXA-193*), and tetracycline (*tetO*) were predominant (*n* = 8/56 isolates; Supplementary Table 7). Interestingly, *gyrA_T86I* and *tetO* were more likely detected in the *C. coli* isolates collected in the winter (60% confidence level and lift of 2), while *aadE-CC* was more likely detected in the *C. coli* isolates collected in the summer (57% confidence level and lift of 2.1). A similar trend was detected with *blaOXA-193* in the summer with *C. jejuni* (40% confidence level and lift of 1.2).

**Table 6:** Antimicrobial resistance genes in thermophilic *Campylobacter*.

Class	Sub-class	Gene	*Campylobacter coli* (*n* = 13)	*Campylobacter jejuni* (*n* = 56)
Aminoglycoside	Amikacin	***aph3’-IIIa***	0%	20%
	Kanamycin			
	Streptomycin	***aadE-Cc***	54%	0%
		***rpsL_K88R***	0%	4%
Beta-lactam	Beta-lactam	***blaOXA-193***	100%	57%
		***blaOXA-449***	0%	5%
		***blaOXA-461***	0%	9%
		***blaOXA-603***	0%	5%
		***blaOXA-61***	0%	2%
Macrolide	Macrolide	***50S_L22_A103V***	0%	14%
Quinolone	Quinolone	***gyrA_T86I***	77%	11%
Tetracycline	Tetracycline	***tetO***	38%	59%

Percentage indicates the prevalence of the designated resistance genes within *Campylobacter jejuni* (*n* = 56) and *Campylobacter coli* (*n* = 13) isolates.

### Co-occurrence of genes from the variable genomes of *Campylobacter* isolates

**Figure 5:**
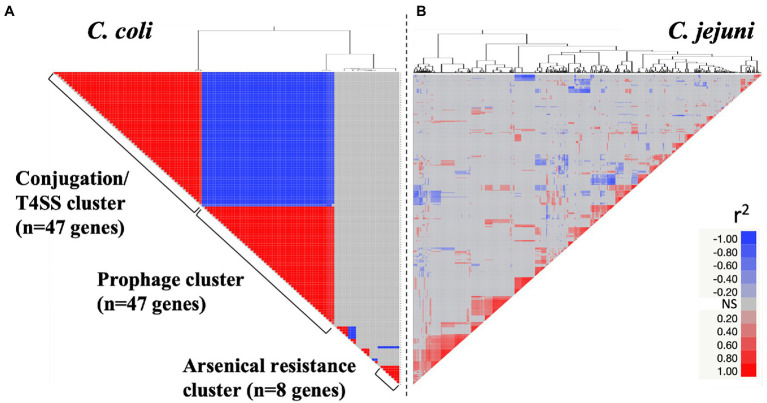
Co-occurrence of genes from the variable genome for both *Campylobacter coli* (**A**; *n* = 102 genes) and *Campylobacter jejuni* (**B**; *n* = 401 genes). X- and Y-axes display the gene co-occurrence profile (r^2^ value). Red and blue cells represent positive and negative correlations between genes of the variable genome (*P* < 0.01), respectively. Gray cells indicate non-significant correlations (NS; *P* > 0.01). Details about the gene co-occurrence profile for *C. coli* are displayed in Supplementary Table 8.

The co-occurrence of genes from the variable genome was investigated in both *C. jejuni* (*n* = 401 genes) and *C. coli* (*n* = 102 genes). Overall, the *C. coli* variable genome was divided into three major clusters with co-occurring genes (Figure 5A; *p* < 0.01). The first co-occurring cluster named “conjugation/T4SS” (r^2^ > 0.8; *n* = 47 genes; Supplementary Table 8) was composed of genes involved in conjugative transfer and T4SS (*n* = 17 genes), capsule associated genes (*n* = 5), *cag12* (encoding cag pathogenicity island protein) and other virulence genes (e.g., *rfbA*, *rfbB,* and *rhmA*). *Campylobacter coli* isolates possessing the *tetO* gene, conferring tetracycline resistance, also possessed genes associated with conjugation/T4SS (r^2^ = 1; *p* < 0.001). A similar observation was made regarding *C. jejuni* (r^2^ > 0.43; *p* < 0.01). Interestingly, *C. coli* isolates possessing these conjugation/T4SS associated genes were less likely to possess prophages (*n* = 22 genes), and virulence genes (e.g., *ceuB*, *fabG, glxK, rfbC*, and *rfbF*; r^2^ < −0.91; *p* < 0.00 l *n* = 47 genes; Figure 5A; Supplementary Table 8). The opposite trend between the co-occurrence of prophage and conjugative/T4SS clusters was observed in *C. jejuni* (r^2^ = 0.69; *p* < 0.01). Both *C. coli* and *C. jejuni* isolates possessing genes implicated in O-methyl phosphoramindate capsule modification (*hddA*, *hddC,* and *hddD*) were also more likely to possess prophages (r^2^ = 0.78 in *C. coli* and r^2^ = 0.37 in *C. jejuni*; *p* < 0.01). *Campylobacter coli* isolates possessing genes associated with arsenical resistance (*n* = 3 genes) were likely to have genes involved in streptomycin resistance (*n* = 1 gene) and other genes (e.g., *yrrC, yraQ* family and *ydeQ*/*yrkL*/*ywrO* family). Unlike *C. coli*, the *C. jejuni* variable genome displayed less pronounced and indistinct gene co-occurrence profiles (Figure 5B).

### Plasmid composition

While no plasmids were detected by PlasmidFinder, MOB-Suite identified a total of 223 plasmids across 69 assembled *Campylobacter* genomes. Only five plasmids were detected in *C. coli* (on average 0.54 plasmids per assembly), whereas 216 plasmids were detected in *C. jejuni* (on average 3.86 plasmids per assembly). Multiple plasmids were detected in each farm. Most plasmids were highly similar and grouped into a total of eight clusters (an approximation of Operational Taxonomic Units for plasmids) and only 18 unique reference plasmids were the nearest neighbors to each detected plasmid (Supplementary Figure 4; Supplementary Table 9). However, it is important to mention that sequencing was conducted using a protocol designed to extract genomic DNA and not plasmid DNA. By consequence, results presented in this section might underestimate the plasmid composition in the *Campylobacter* isolates. Overall, among the 18 plasmids, 10 of them harbored ARG associated with the resistance to tetracyclines and aminoglycosides, four of them harbored genes associated with the T6SS and two other plasmids harbored genes associated with the T4SS. The prevalence of certain plasmids was closely associated with the ST-CC profile of the isolates.

## Discussion

This study further demonstrates that dairy manure is a potential reservoir of thermophilic *Campylobacter* and a possible mode of transmission of *Campylobacter* to the human food chain (An et al., 2018; Sheng et al., 2019; Szott and Friese, 2021). In this study, whole genome sequencing (WGS) was conducted to identify the *Campylobacter* isolates obtained from the dairy manure used in SSCF as well as to better understand the diversity of genomic traits in the isolates. WGS demonstrated that *C. jejuni* (*n* = 56/69) was more predominant in dairy manure compared to *C. coli* (*n* = 13/69), which mirrors previous findings (Ocejo et al., 2019; Hansson et al., 2020). Overall, *Campylobacter* was recurrently detected in the SSCF between 2018 and 2020. Distinct genomic profiles were observed between *C. coli* and *C. jejuni* isolates, independent of the location and time of collection. Furthermore, the variable genomes of the *Campylobacter* isolates were closely associated with their ST and CC profile. Based on SNP profiling and MLST data, *Campylobacter* isolates collected from the same SSCF were more likely to belong to the same CC and harbor high genomic content similarity compared to isolates from other SSCF; Nevertheless, genomic profile similarities were also detected to some extent between SSCF. Thereby, our data suggest that *Campylobacter* may persist for an extended period of time within a cattle herd of a SSCF and could be transmitted between SSCF. We hypothesize that the dissemination of *Campylobacter* within or between the SSCFs could be due to sharing of agricultural equipment or fields. However, our current data does not allow determination as to whether (1) the infected cattle shed *Campylobacter* for an extended period, (2) *Campylobacter* persist in the SSCF by re-infecting cattle at different times over the course of the year, (3) the same *Campylobacter* isolates are re-introduced into the farms over time through interactions with the ecosystem surrounding the farm, or (4) how many cattle within the herd are infected with *Campylobacter*.

Distinct differences in functional genomics (gene content, virulome, and resistome) were observed between and within *Campylobacter* species and were also influenced by the spatial distribution of the SSCF where the isolates were collected. Several pathways associated with the capsule modification/biosynthesis, sugar synthesis/utilization, conjugative transfer, T4SS, and prophage diversity were associated with specific CC-ST profiles (Tables 4, 5). The main variations in gene content detected between isolates were related to gene transfer such as conjugative transfer (IncQ/IncF) and T4SS, which are key bacterial mechanisms involved in the virulence of *Campylobacter* and in the survival in specific environmental niches (Bacon et al., 2000; Huddleston, 2014; Deblais et al., 2018; Marasini et al., 2020; Panzenhagen et al., 2021). *Campylobacter coli* (*n* = 5/13) and *C. jejuni* (*n* = 22/59) isolates recovered in this study possessed several IncQ/IncF (*traB,C,E,G,H,K,L,N,Q,R,T,U,V,* and *W*) and T4SS (*virB2-11* and *virD4*) protein-encoding genes. The presence of these genes was previously reported to be rare in *C. jejuni* and *C. fetus* subsp. *venerealis* isolates worldwide (Bacon et al., 2000; Panzenhagen et al., 2021; Silva et al., 2021). A mutation in *virB11* was also shown to reduce *C. jejuni* 81–176 adhesion and invasion by 6- and 11-fold, respectively compared to wild-type (Bacon et al., 2000). Conjugation/T4SS-associated genes are frequently detected in *Campylobacter* isolated from livestock and are transmitted by plasmids also carrying ARG (Marasini et al., 2018, 2020; Ocejo et al., 2019). Similar trends were observed in our study. The resistance to tetracycline (*tetO*) and amikacin/kanamycin (*aph3’-IIIa*) in both *C. jejuni* and *C. coli* were highly correlated with the presence of IncQ-IncF/T4SS genes (r^2^ > 0.5; *p* < 0.001). Similarly, the gene encoding the Cag12 pathogenicity island protein was also highly correlated with the genes mentioned above (r^2^ > 0.8; *p* < 0.001). Overall, our study highlights that thermophilic *Campylobacter* recovered from SSCF carry plasmid-encoded proteins essential for its virulence (Bacon et al., 2000; Marasini et al., 2018, 2020). Based on these observations, DNA extraction using protocols allowing for the isolation of both genomic and plasmid DNA will be used for future studies to enhance the resolution of the genomic interpretations and better understand horizontal transfer dynamics. Preliminary data showed that plasmids associated with tetracycline resistance, the T4SS, and the T6SS were predominant, especially in *C. jejuni* isolates. Similar plasmids (e.g., plasmid accession number CP023447) were previously reported in *C. jejuni* isolated from poultry meat in Brazil (de Fátima Rauber Würfel et al., 2020). However, it is important to mention that the DNA extraction method used in this study limited the in-depth analysis and resolution of the plasmid composition in the *Campylobacter* isolates.

Interestingly, the presence of genes involved in DNA/protein transfer was negatively correlated with the prevalence of prophages in *C. coli* genomes, while the opposite trend was observed in *C. jejuni*. Bacteriophages/prophages have been linked to the acquisition of novel host survival strategies, and virulence and antimicrobial resistance genes (Kondo et al., 2021; Torres-Barceló, 2018; Pratama and van Elsas, 2019). Similarly, there were strong, negative correlations of bacteriophages/prophages with certain protein-encoded genes associated with O-methyl phosphoramidate modification of the capsule (gamma-glutamyl-CDP-amidate hydrolase, L-glutamine kinase, methyltransferase [EC 2.1.1.-], and phosphoglutamine cytidylyltransferase [EC 2.7.7.-]), and the utilization of UDP-activated sugars (UDP-glucose, UDP-galactopyranose and UDP-glucuronate, and L-glutamine kinase) in both *C. coli* and *C. jejuni*. Other genes implicated in O-methyl phosphoramidate capsule modification (*hddA*, *hddC,* and *hddD*) displayed the opposite trend, which follow previously published data concerning the role of capsule polysaccharides in phage sensitivity (Cai et al., 2019). A previous study demonstrated that the modification of the capsule polysaccharides modulates the phage infectivity in *C. jejuni* (Sørensen et al., 2012). Further, it was hypothesized that the level of UDP-activated sugars may be associated with the cell wall integrity due to phage infection (Ankrah et al., 2014). Interestingly, similar trends concerning the composition of conjugation/T4SS genes and prophages were observed with *Salmonella enterica* subsp. *enterica* serotype Heidelberg isolates collected from poultry farms in the Midwest (Deblais et al., 2018). Thus, our data suggests the importance of conjugation/T4SS genes and prophages in the genome plasticity as well as the emergence of antibiotic resistance, survival, and virulence abilities in thermophilic *Campylobacter*.

The prevalence of ARGs for aminoglycoside, beta-lactam, quinolone, and tetracycline was predominant (>29%) in *Campylobacter* isolates in this study. Similar trends were observed in published studies (Ocejo et al., 2019; Cobo-Díaz et al., 2021; Hailu et al., 2021; Hull et al., 2021). As previously described (Ocejo et al., 2019; Mouftah et al., 2021), *blaOXA-193* was the most predominant ARG to beta-lactam compared to other beta-lactamase encoding genes (*blaOXA-449, -461, -603,* and *61*; <10%). However, some other studies have reported *blaOXA-61* being predominant in *Campylobacter* (Cobo-Díaz et al., 2021; Hull et al., 2021). Such discrepancies could be associated with annotation errors, which may lead to wrongly identifying *bla-OXA-193* as *bla-OXA-61* (Feldgarden et al., 2019b). It was previously shown that human contact with cattle could be linked to the transmission of tetracycline resistant *C. jejuni* (ST-464, ST-459, and ST-982; Cha et al., 2016, 2017). However, in the current study, resistance to tetracycline was broadly distributed across 12 other STs, especially in CC-21 (*n* = 25/56) which is the most predominant CC identified similar to a previously published study (An et al., 2018). CC-828 was the most prevalent clonal complex detected among the *C. coli* isolates (*n* = 13/13), which agrees with previous studies performed on ruminants (especially sheep) from Nigeria, Scotland, and Spain (Sproston et al., 2011; Ngulukun et al., 2016; Ocejo et al., 2019). The predominance of CC-21 and CC-828 in the dairy manure collected in SSCF from Northeast Ohio, United States is expected given they are known host generalist clonal complexes (Ocejo et al., 2021). Other STs (ST-829) were previously detected in chicken farms and slaughter houses, suggesting that horizontal transmission of *Campylobacter* may occur between livestock species (Frosth et al., 2020). ST-8 was previously isolated from sheep and cattle and was associated with abortion (Sahin et al., 2008; Wu et al., 2014; Tang et al., 2017).

Data obtained in this study showed that *C. jejuni* isolated from dairy manure harbored genes associated with legionaminic acid, glutamate/fucose, and pantothenic acid (vitamin B5) metabolism. It was previously reported that vitamin B5 biosynthesis enhances *C. jejuni* colonization of cattle and was associated with human infections (Sheppard et al., 2013; Buchanan et al., 2017). Further, the competition for vitamin B5 between *Campylobacter* and the host may affect the host immune system and increase susceptibility to other pathogens (Yoshii et al., 2019). Legionaminic acid is involved in the modification of bacterial flagellin which is key for the persistence of *Campylobacter* in poultry and host interactions (Howard et al., 2009; Stephenson et al., 2014; Ardissone et al., 2020; Vieira et al., 2021); however, its role in the persistence of *Campylobacter* in cattle and dairy manure remains unknown. The utilization of fucose in nutrient-limited conditions has been reported to provide a competitive advantage to *C. jejuni* by enhancing cell adhesions and biofilm production (Day et al., 2009; Muraoka and Zhang, 2011; Stahl et al., 2011; Dwivedi et al., 2016). Similar observations were reported regarding the vital role of glutamate in *C. jejuni* metabolism and as a chemoattractant (Lübke et al., 2018). However, it is important to mention that most of these studies have been performed in poultry.

## Conclusion

In conclusion, our study demonstrated that several thermophilic *Campylobacter* STs isolated from dairy manure in SSCF from Northeast Ohio were previously associated with human campylobacteriosis cases, and thus, signals public health risks associated with the presence of dairy cattle in these mixed farming systems. Furthermore, the thermophilic *Campylobacter* isolates studied frequently harbored ARGs related to quinoline, beta-lactam, tetracycline, and streptomycin resistance. Our data also highlights potential risks associated with the long-term persistence of thermophilic *Campylobacter* within the same SSCF farm over time. Similarly, our findings suggest potential interactions between farms leading to the dissemination of thermophilic *Campylobacter* isolates between farms of proximity. Therefore, an emphasis on the understanding of SSCF practices and potential interactions between SSCF will help in identifying factor(s) that influence the persistence and dissemination of *Campylobacter* within and between SSCF, and thus, develop new guidelines to mitigate *Campylobacter* burden in SSCF.

## Data availability statement

The datasets presented in this study can be found in online repositories. The names of the repository/repositories and accession number(s) can be found in the article/Supplementary material.

## Author contributions

LD, MK, HJ, KB, UB, LH, KH, and GR: experimental designing. MK: collection and processing of the samples. MK, HJ, MS, and SB: extraction of DNA and preparation of samples for sequencing. LD, MK, GR, HJ, KB, JP, and JG: analyzing and interpretation of sequencing results. LD, GR, HJ, JG, and KB: writing the manuscript. All authors contributed to the article and approved the submitted version.

## Funding

This research was supported by state and federal funds appropriated to the Ohio Agricultural Research and Development Center, The Ohio State University. Funds for sequencing was made available by the Produce Safety Research Consortium, CFSAN, FDA. MS and SB are Fellows supported by Goldbelt C6, LLC.

## Conflict of interest

The authors declare that the research was conducted in the absence of any commercial or financial relationships that could be construed as a potential conflict of interest.

## Publisher’s note

All claims expressed in this article are solely those of the authors and do not necessarily represent those of their affiliated organizations, or those of the publisher, the editors and the reviewers. Any product that may be evaluated in this article, or claim that may be made by its manufacturer, is not guaranteed or endorsed by the publisher.

## Footnotes

^1^ https://github.com/aldertzomer/cgmlst

^2^ https://pubmlst.org/organisms/campylobacter-jejunicoli
