# Supplementary material for: Whole genome characterization of thermophilic Campylobacter species isolated from dairy manure in small specialty crop farms of Northeast Ohio
Source: Front Microbiol. 2023 Mar 21;14:1074548. doi: 10.3389/fmicb.2023.1074548 (PMC10071015; doi:10.3389/fmicb.2023.1074548)
Supplement: Supplementary file 1 [file Data_Sheet_1.pdf]

Supplementary Material

Supplemental Table 1. Whole genome sequencing results of the thermophilic *Campylobacter* isolates. N=13 *C. coli* and 56 *C. jejuni* isolates.

***Campylobacter* species**	***C. coli* (mean[IC95%])**	***C. jejuni* (mean[IC95%])**
Number of contigs	44 [19-69]	39 [35-43]
Length (bp)	1,691,610 [1,680,103-1,703,117]	1,683,112 [1,664,247-1,701,977]
N50	138,085 [105,915-170,255]	132,935 [115,659-150,212]

Supplemental Table 2. Details about the *Campylobacter* isolates.

Species	NCBI ID	Year of collection	Month of collection	Farm ID	Farm cluster	ST	CC	# contigs	Genome size	Sequencing depth	N50	Mean Length_R1	Mean Length_R2	Mean Q_R1	Mean Q_R2	Number of CDS
Cc	112127	2019	12	8	2	1068	828	37	1712414	258	130748	158	158	36.5	36.3	1828
Cj	112130	2019	12	9	3	403	403	41	1730330	159	159682	207	207	36.7	35.9	1904
Cc	112141	2020	3	7	1	1068	828	26	1712508	194	177425	208	209	36.6	35.6	1837
Cc	112142	2020	3	8	2	1068	828	29	1712828	258	162623	190	190	36.8	36.2	1838
Cj	112146	2020	5	9	3	464	464	41	1690190	204	118700	224	224	37	36	1871
Cc	112149	2020	5	7	1	829	828	31	1685584	212	149009	218	218	36.9	35.9	1823
Cj	112152	2020	5	8	2	8	21	59	1677351	166	174488	203	204	36.8	35.9	1812
Cc	112154	2020	5	8	2	1068	828	28	1714190	213	162597	226	227	36.9	35.5	1835
Cc	112156	2020	5	34	5	1068	828	29	1712000	273	238715	215	215	36.9	35.9	1830
Cc	112158	2020	5	7	1	829	828	31	1683322	209	159067	184	184	36.9	36.7	1819
Cj	112159	2020	5	8	2	8	21	48	1650334	73.4	74419	206	206	36.5	36	1796
Cc	112160	2020	5	34	5	829	828	42	1670714	105	96671	174	174	36.8	36.2	1809
Cc	112161	2020	6	7	1	829	828	31	1683677	71.5	141590	181	181	36.6	35.9	1816
Cj	112164	2020	6	8	2	929	21	49	1658617	82.9	79572	203	203	36.7	36.3	1820
Cj	112166	2020	7	8	2	929	21	42	1655476	64.8	82666	194	194	36.7	36.3	1814
Cj	112167	2020	7	8	2	929	21	69	1662280	85.4	79076	151	151	36.5	36.2	1819
Cc	112171	2020	7	7	1	829	828	50	1680862	73.6	87226	191	191	36.8	36.3	1826
Cc	112172	2020	7	8	2	829	828	179	1656321	24.5	15642	178	178	36.6	35.5	1860
Cc	112173	2020	7	34	5	829	828	21	1683966	131	161488	191	192	36.9	35.3	1818
Cj	112177	2020	8	7	1	5261	257	50	1762741	232	105461	157	157	36.7	36	1964
Cj	112180	2020	8	8	2	8	21	31	1651930	182	174548	181	181	36.9	36.8	1794
Cc	112184	2020	8	7	1	829	828	39	1682555	216	112311	185	185	36.9	36.4	1824
Cj	112913	2018	1	1	1	11771	42	33	1762271	118	114572	195	196	36.2	33.8	1925
Cj	112917	2018	2	1	1	267	283	32	1642110	182	129135	194	195	36.5	33.9	1742
Cj	112921	2018	3	19	5	48	48	34	1710031	153	116332	198	199	36.5	34.2	1867
Cj	112922	2018	3	17	2	8	21	30	1615799	167	182447	170	173	36.4	32.7	1736
Cj	112924	2018	3	19	5	21	21	63	1757349	151	90220	189	191	36.4	32.3	1937
Cj	112925	2018	4	11	4	42	42	50	1657537	173	148161	190	190	36.4	36.3	1821
Cj	112926	2018	4	11	4	922	21	27	1634735	87.8	197356	209	209	35.9	35.9	1743
Cj	112927	2018	5	1	1	459	42	36	1800378	124	108902	162	163	36.3	35.4	1978
Cj	112930	2018	5	7	1	806	21	92	1873465	167	60119	154	154	36.2	35.3	2116
Cj	112932	2018	5	17	2	8	21	39	1614640	126	98111	172	172	36.2	36.2	1745

Cj	112935	2018	7	1	1	922	21	43	1637610	163	92304	181	181	36.3	36.1	1751
Cj	112936	2018	7	9	3	806	21	52	1823936	145	85847	163	163	36.3	35.2	2051
Cj	112944	2019	1	17	2	459	42	52	1765194	114	103258	174	174	36.3	35.2	1941
Cj	112949	2019	2	26	1	922	21	68	1686980	142	98215	167	168	36.2	34	1804
Cj	112953	2019	2	30	3	61	61	18	1611071	98.3	377770	175	179	36.4	31.9	1753
Cj	112957	2019	2	11	4	52	52	55	1598509	195	98736	163	167	36.1	32.4	1739
Cj	112958	2019	2	26	1	922	21	59	1681245	171	95146	200	200	36.4	36.3	1814
Cj	112960	2019	2	17	2	459	42	34	1768738	146	114505	202	202	36.5	35.9	1950
Cj	112961	2019	3	26	1	922	21	48	1682222	243	99116	202	202	36.6	36.2	1806
Cj	112962	2019	3	30	3	61	61	18	1612444	230	168783	186	187	36.5	36.2	1757
Cj	112967	2019	3	7	1	45	45	27	1637612	116	136755	200	200	36.4	35.7	1713
Cj	112968	2019	3	30	3	45	45	34	1634642	195	127155	181	181	36.5	35	1713
Cj	112969	2019	3	7	1	42	42	22	1650130	56.2	155132	217	217	36.3	34.1	1793
Cj	112970	2019	3	26	1	922	21	28	1637177	162	447899	208	208	36.5	35.8	1750
Cj	112971	2019	4	17	2	1244	61	27	1708668	131	116155	215	216	36.7	35.7	1877
Cj	112974	2019	4	26	1	922	21	30	1635508	167	154529	220	220	36.6	35.4	1750
Cj	112979	2019	4	11	4	52	52	33	1599234	154	181186	222	222	36.5	35.5	1725
Cj	112980	2019	4	26	1	52	52	32	1598761	162	118671	224	224	36.6	34.9	1722
Cj	112981	2019	5	17	2	459	42	35	1769103	103	114386	228	228	36.5	34.8	1948
Cj	112985	2019	5	30	3	21	21	28	1663450	89.4	147015	221	221	36.3	35.7	1811
Cj	112990	2019	5	7	1	61	61	17	1611513	82.3	122876	221	221	36.1	35.8	1749
Cj	112991	2019	5	30	3	61	61	27	1611575	163	154933	158	159	36.4	35.9	1741
Cj	112992	2019	6	17	2	806	21	41	1849238	129	98715	192	192	36.6	35.9	2072
Cj	112998	2019	6	26	1	45	45	36	1634818	182	107481	167	167	36.4	36.5	1714
Cj	112999	2019	7	7	1	922	21	35	1634014	163	162464	179	179	36.6	36.5	1747
Cj	113000	2019	7	26	1	604	42	51	1627489	122	53847	192	192	36.5	36.1	1756
Cj	113001	2019	7	30	3	61	61	25	1610973	98.9	109892	186	186	36.5	36.1	1755
Cj	113002	2019	7	26	1	922	21	42	1681246	80.2	107561	211	211	36.5	35.3	1799
Cj	113003	2019	7	30	3	61	61	18	1611373	118	175368	158	159	36.4	35.1	1743
Cj	113004	2019	8	17	2	982	21	36	1695487	261	109963	178	179	36.5	35.7	1859
Cj	113005	2019	8	26	1	982	21	29	1695774	167	115645	195	195	36.6	36.4	1858
Cj	113007	2019	8	30	3	61	61	30	1610024	196	107152	196	196	36.6	35.9	1758
Cj	113015	2019	9	26	1	922	21	41	1681228	378	149097	186	186	36.6	36.2	1800
Cj	113016	2019	9	17	2	806	21	41	1827031	121	154473	222	222	36.6	35.7	2054
Cj	113225	2020	10	8	2	403	403	33	1733602	218	192307	211	212	36.9	36.4	1908
Cj	113229	2020	10	7	1	5261	257	32	1761187	214	107458	182	182	37	36.7	1965

Cj	113230	2020	10	8	2	11712	42	41	1767923	134	88638	212	212	37	36.2	1947

Cj and *Cc*: *Campylobacter jejuni* and *coli*, respectively. CC: clonal complex. ST: sequence type. N50: sequence length of the shortest contig at 50% of the total genome length. Mean Q: average base calling accuracy. R1 and R2: forward and reverse reads, respectively. CDS: coding sequences.

Supplemental Table 3. Protein encoding genes unique to *Campylobacter jejuni* (n=56 isolates) or *Campylobacter coli* (n=13 isolates).

**Protein-encoding gene function**	**Comment**
Methyl accepting chemotaxis protein	
Methyl accepting chemotaxis sensor/transducer protein	
Putative MCP domain signal transduction protein	
CopG protein	
Periplasmic dsDNA and ssDNA binding protein contributing to transformation	
Osmosensitive K+ channel histidine kinase KdpD	
Potassium transporting ATPase A chain (EC 3.6.3.12) (TC 3.A.3.7.1)	
Potassium transporting ATPase B chain (EC 3.6.3.12) (TC 3.A.3.7.1)	
Potassium transporting ATPase C chain (EC 3.6.3.12) (TC 3.A.3.7.1)	
RepA protein homolog	
Inosine uridine preferring nucleoside hydrolase (EC 3.2.2.1)	
Major antigenic peptide PEB3	
Small hydrophobic protein Cj0260c	
N linked glycosylation glycosyltransferase PglG	
Transcriptional regulator, HxlR family	
Putative secreted serine protease (EC 3.4.21.)	
serine protease pet	
Beta 1,4 N acetylgalactosaminyltransferase (EC 2.4.1.)	
2 oxoglutarate/malate translocator	
Gluconate 2 dehydrogenase (EC 1.1.99.3), membrane bound, flavoprotein	
Gluconate 2 dehydrogenase (EC 1.1.99.3), membrane bound, gamma subunit	
Surface exposed lipoprotein JlpA	Genes only detected in *Campylobacter jejuni* isolates
CRISPR associated endonuclease Cas9	
CRISPR associated protein Cas1	

CRISPR associated protein Cas2	
Cytochrome c family protein	
Heterodisulfide reductase subunit B like protein @ Putative succinate dehydrogenase subunit	
Succinate dehydrogenase flavoprotein subunit (EC 1.3.5.1)	
Succinate dehydrogenase iron sulfur protein (EC 1.3.5.1)	
putative molybdenum containing oxidoreductase	
Probable sulfite oxidase	
putative 2 pyrone 4,6 dicarboxylic acid hydrolase	
Putative arylsulfate sulfotransferase (EC 2.8.2.22)	
Hippurate hydrolase (EC 3.5.1.32)	
RidA/YER057c/UK114 superfamily, group 2, YoaB	
Cj0990c	
jhp1395	
Cj0186c Integral membrane protein TerC	
LSU ribosomal protein L36p @ LSU ribosomal protein L36p, zinc dependent	
Citrate/H+ symporter of CitMHS family	
CoA binding domain protein	
Di tripeptide/cation symporter	
DNA transfer protein	
Putative processing peptidase	
Family of unknown function (DUF450) family	
FIG001614: Membrane protein	
FIG00470841: hypothetical protein	
FIG015373: Membrane protein	
Arsenate reductase (EC 1.20.4.4) thioredoxin-coupled, LMWP family	Genes only detected in *Campylobacter coli* isolates
Arsenical-resistance protein ACR3	
probable periplasmic protein Cj0413 , putative	
Penicillin-insensitive transglycosylase (EC 2.4.2.-) & transpeptidase PBP-1C	

Conjugative transfer protein TrbL	
Sulfate adenylyltransferase subunit 1 (EC 2.7.7.4) / Sulfate permease, Trk-type	
Sulfate adenylyltransferase subunit 2 (EC 2.7.7.4)	
Tricarboxylate transport protein TctB	
Tripartite tricarboxylate transporter TctA family	
Tripartite tricarboxylate transporter TctC family	
Capsular polysaccharide synthesis protein	
Cell wall-associated hydrolase	
Protein-methionine-sulfoxide reductase catalytic subunit MsrP	
Putative polysaccharide deacetylase	
Putative methyl-accepting chemotaxis protein	
SAM-dependent methyltransferase	
lipopolysaccharide core biosynthesis protein LpsA	
UPF0053 inner membrane protein YgdQ; S-adenosylmethionine synthetase (Methionineadenosyltransferase AdoMet synthetase MAT)	
UDP-glucose 6-dehydrogenase (EC 1.1.1.22)	
2-methylcitrate dehydratase (EC 4.2.1.79)	
2-methylcitrate synthase (EC 2.3.3.5)	
4-cresol dehydrogenase [hydroxylating] flavoprotein subunit (EC 1.17.99.1)	
Adenine-specific DNA methyltransferase	
Adenylylsulfate kinase (EC 2.7.1.25)	
AgrC, putative	
Aliphatic amidase AmiE (EC 3.5.1.4)	
Metal chaperone, involved in Zn homeostasis	
Peroxide stress regulator / Ferric uptake regulation protein	
Uncharacterized metal ion transporter YcsG, Mn(2+)/Fe(2+) NRAMP family	
sarcosine oxidase, putative	
Methylisocitrate lyase (EC 4.1.3.30)	
Alternative cytochrome c oxidase polypeptide CoxO (EC 1.9.3.1)	

sodium/pantothenate symporter , putative	
UDP-glucuronate decarboxylase (EC 4.1.1.35)	
Channel-forming transporter/cytolysins activator of TpsB family	
DnaJ-related protein	
Hemerythrin-like iron-binding protein	
L-carnitine dehydratase/bile acid-inducible protein F	
Methionine synthase II (cobalamin-independent)	
Permease of the drug/metabolite transporter (DMT) superfamily	
Phosphonate ABC transporter phosphate-binding periplasmic component (TC 3.A.1.9.1)	
Putative large exoprotein involved in heme utilization or adhesion of ShlA/HecA/FhaA family	
Putative MCP-type signal transduction protein	
Tat (twin-arginine translocation) pathway signal sequence domain protein	
integral membrane protein	
integral membrane protein, putative	
Citrate lyase beta chain (EC 4.1.3.6)	
Inner membrane protein CreD-like protein	
Inner membrane protein YihN	
Inner membrane protein YihY, formerly thought to be RNase BN	
probable integral membrane protein Cj0564 - related protein	
Acetate permease ActP (cation/acetate symporter)	
AzlC family protein	
Branched-chain amino acid transport protein, putative	
CAAX amino terminal protease family protein	
COGs COG3777	
Dienelactone hydrolase and related enzymes	
glcG protein	
Hemolysin, putative	
Leader peptidase (Prepilin peptidase) (EC 3.4.23.43)	

major facilitator family transporter, putative	
membrane protein	
Propionate--CoA ligase (EC 6.2.1.17); Acetyl-CoA synthetase (EC 6.2.1.1)	
RecD-like DNA helicase YrrC	
Ribosomal RNA adenine dimethylase	
Tgh102	
TM2 domain family	
transcriptional regulator, Crp/Fnr family	
Uncharacterized NAD(P)H oxidoreductase, YdeQ/YrkL/YwrO family	
UPF0033 protein YeeD; Putative sulfur carrier protein YeeD	
UPF0317 protein YcsI	
UPF0394 inner membrane protein YeeE	
FIG00469465: hypothetical protein	
FIG00469601: hypothetical protein	
FIG00469817: hypothetical protein	
FIG00469900: hypothetical protein	
FIG00469923: hypothetical protein	
FIG00470545: hypothetical protein	
FIG00470755: hypothetical protein	
FIG00472317: hypothetical protein	
FIG00710175: hypothetical protein	

Supplemental Table 4. Protein encoding genes belonging to *Campylobacter jejuni* (n=56 isolates) variable genomes.

**Cluster**	**Protein-encoding gene**
Cj1	Beta-1,4-N-acetylgalactosaminyltransferase (EC 2.4.1.-)
Cj1	Biotin sulfoxide reductase (EC 1.-.-.-) / Free methionine-(S)-sulfoxide reductase
Cj1	Cytidine diphosphoramidate kinase Cj1415
Cj1	FIG00469638: hypothetical protein
Cj1	gamma-Glutamyl-CDP-amidate hydrolase involved in O-methyl phosphoramidate capsule modification
Cj1	Homolog of BLC protein
Cj1	L-glutamine kinase involved in O-methyl phosphoramidate capsule modification
Cj1	Methyltransferase (EC 2.1.1.-), possibly involved in O-methyl phosphoramidate capsule modification
Cj1	Peptide-methionine (R)-S-oxide reductase MsrB (EC 1.8.4.12)
Cj1	Phosphoglutamine cytidylyltransferase (EC 2.7.7.-) in O-methyl phosphoramidate capsule modification
Cj1	Phosphoglycerol transferase-like protein
Cj1	Predicted D-lactate dehydrogenase, Fe-S protein, FAD/FMN-containing
Cj1	Predicted glycosyltransferase involved in capsule biosynthesis
Cj1	Pseudaminic acid biosynthesis protein PseA, possible Pse5Ac7Ac acetamidino synthase
Cj1	putative cytochrome C-type haem-binding periplasmic protein
Cj1	Similar to imidazole glycerol phosphate synthase amidotransferase subunit (LPS cluster)
Cj1	Similar to imidazole glycerol phosphate synthase cyclase subunit (LPS cluster)
Cj1	Zinc transporter ZupT
Cj2	3-oxoacyl-[acyl-carrier-protein] synthase, KASIII (EC 23141)
Cj2	Anaerobic dimethyl sulfoxide reductase chain A (EC 1853), molybdopterin-binding domain
Cj2	Anaerobic dimethyl sulfoxide reductase chain B (EC 1853), iron-sulfur binding subunit
Cj2	Anaerobic dimethyl sulfoxide reductase chain C (EC 1853), anchor subunit
Cj2	Anaerobic dimethyl sulfoxide reductase chaperone DmsD
Cj2	CDP-4-dehydro-6-deoxy-D-glucose 3-dehydratase (EC 421-)
Cj2	death-on-curing family protein

Cj2	FIG00469988: hypothetical protein
Cj2	FIG00470572: hypothetical protein
Cj2	FIG00472892: hypothetical protein
Cj2	Hypothetical protein Cj1300
Cj2	methyltransferase FkbM family
Cj2	Oxidoreductase, aldo/keto reductase family
Cj2	Tgh088
Cj2	TraG-like protein
Cj2	UDP-N-acetyl-D-mannosamine dehydrogenase (EC 111336)
Cj2	UDP-N-acetylglucosamine 2-epimerase (EC 51314)
Cj2	Uncharacterized protein ImpH/VasB
Cj3	3-methyl-2-oxobutanoate hydroxymethyltransferase (EC 2.1.2.11)
Cj3	ABC transporter, permease protein 1 (cluster 1, maltose/g3p/polyamine/iron)
Cj3	ABC transporter, substrate-binding protein (cluster 1, maltose/g3p/polyamine/iron)
Cj3	Aspartate 1-decarboxylase (EC 4.1.1.11)
Cj3	Bacteriocin resistance protein; peptidase C39
Cj3	D-glycero-alpha-D-manno-heptose 1-phosphate guanylyltransferase (EC 2.7.7.71)
Cj3	D-glycero-alpha-D-manno-heptose 7-phosphate kinase (EC 2.7.1.168)
Cj3	dTDP-4-dehydrorhamnose 3,5-epimerase (EC 5.1.3.13)
Cj3	Ferric iron ABC transporter, ATP-binding protein
Cj3	FIG00973752: TolA-like membrane protein
Cj3	GDP-L-fucose synthetase (EC 1.1.1.271)
Cj3	GDP-mannose 4,6-dehydratase (EC 4.2.1.47)
Cj3	Pantoate--beta-alanine ligase (EC 6.3.2.1)
Cj3	Putative hemolysin activation/secretion protein
Cj3	Putative secreted serine protease (EC 3.4.21.-)
Cj3	serine protease pet
Cj4	3',5'-cyclic-nucleotide phosphodiesterase (EC 3.1.4.17)

Cj4	Arsenate reductase (EC 1.20.4.4) thioredoxin-coupled, LMWP family
Cj4	Arsenical-resistance protein ACR3
Cj4	Class D beta-lactamase (EC 3.5.2.6) => OXA-184 family
Cj4	FIG00471540: hypothetical protein
Cj4	FIG00471788: hypothetical protein
Cj4	Glycerol-3-phosphate ABC transporter, periplasmic glycerol-3-phosphate-binding protein (TC 3.A.1.1.3)
Cj4	HAD-superfamily hydrolase, subfamily IA, variant 1
Cj4	Histidinol-phosphate aminotransferase (EC 2.6.1.9)
Cj4	Hypothetical protein Cj1433c
Cj4	hypothetical protein; Some similarities with formyltransferase
Cj4	Inner membrane protein YihN
Cj4	Methyltransferase, FkbM family protein
Cj4	N-Acetyl-D-glucosamine ABC transport system, permease protein 1
Cj4	Na+/H+ antiporter
Cj4	Putative sugar transferase
Cj4	radical SAM domain protein protein
Cj4	Transcriptional regulator, Xre family
Cj4	Type III restriction-modification system DNA endonuclease res (EC 3.1.21.5)
Cj4	UDP-galactopyranose mutase (EC 5.4.99.9)
Cj4	UDP-glucose 6-dehydrogenase (EC 1.1.1.22)
Cj4	UDP-glucuronate decarboxylase (EC 4.1.1.35)
Cj4	Zn peptidase
Cj5	Ferric siderophore transport system, biopolymer transport protein ExbD
Cj5	FIG00470143: hypothetical protein
Cj5	FIG00545237: hypothetical protein
Cj5	FIG00638667: hypothetical protein
Cj5	FIG00732228: membrane protein
Cj5	GDP-2-acetamido-2,6-dideoxy-alpha-D-xylo-hexos-4-ulose aminotransferase [PLP]

Cj5	GDP-N-acetylglucosamine 4,6-dehydratase [NAD+]
Cj5	Glucosamine-1-phosphate guanylyltransferase
Cj5	Glutamine--fructose-6-phosphate transaminase (isomerizing), glutaminase subunit (EC 26116)
Cj5	Glutamine--fructose-6-phosphate transaminase (isomerizing), isomerase subunit (EC 26116)
Cj5	ISCco1, transposase orfB
Cj5	Legionaminic acid biosynthesis protein PtmG
Cj5	LSU rRNA ## 23S rRNA, large subunit ribosomal RNA
Cj5	N,N'-diacetyllegionaminic acid synthase (EC 251101)
Cj5	Possible restriction /modification enzyme
Cj5	Probable membrane protein Cj0124c
Cj5	Putative H-T-H containing protein
Cj5	Putative integral membrane protein
Cj5	Putative lipoprotein, similar to CjrA of Escherichia coli O164
Cj5	Putative outer membrane siderophore receptor
Cj5	Type I restriction-modification system, DNA-methyltransferase subunit M (EC 21172) / Type I restriction-modification system, specificity subunit S
Cj5	UDP-N,N'-diacetylbacillosamine 2-epimerase (hydrolyzing) (EC 321184)
Cj6	Aldehyde dehydrogenase A (EC 12122) @ Glycolaldehyde dehydrogenase (EC 12121)
Cj6	Altronate dehydratase (EC 4217)
Cj6	Dihydrodipicolinate synthase family
Cj6	FIG00469761: hypothetical protein
Cj6	First ORF in transposon ISC1904
Cj6	Fucose permease
Cj6	L-fuco-beta-pyranose dehydrogenase, type 2 (EC 1111122)
Cj6	L-fuconolactone hydrolase
Cj6	L-fucose mutarotase (EC 51329)
Cj6	Mobile element protein
Cj6	Probable lipoprotein Cj0818-related protein

Cj6	Transcriptional regulator, IclR family
Cj7	4-carboxymuconolactone decarboxylase (EC 4.1.1.44)
Cj7	Beta-1,3-glucosyltransferase
Cj7	Beta-1,4-galactosyltransferase
Cj7	capsular polysaccharide biosynthesis protein, putative
Cj7	conserved hypothetical protein
Cj7	Conserved putative membrane protein precursor
Cj7	DNA methylase N-4/N-6 domain protein
Cj7	dTDP-3-amino-3,6-dideoxy-alpha-D-galactopyranose 3-N-acetyltransferase (EC 2.3.1.197)
Cj7	dTDP-3-amino-3,6-dideoxy-alpha-D-galactopyranose transaminase (EC 2.6.1.90)
Cj7	dTDP-6-deoxy-3,4-keto-hexulose isomerase (EC 5.3.2.3)
Cj7	dTDP-glucose 4,6-dehydratase (EC 4.2.1.46)
Cj7	FIG00470547: hypothetical protein
Cj7	FIG00470593: hypothetical protein
Cj7	FIG00470597: hypothetical protein
Cj7	FIG00470843: hypothetical protein
Cj7	FIG00471415: hypothetical protein
Cj7	FIG00896760: hypothetical protein
Cj7	Glucose-1-phosphate thymidylyltransferase (EC 2.7.7.24)
Cj7	Guanine/hypoxanthine permease
Cj7	Homoserine O-succinyltransferase (EC 2.3.1.46)
Cj7	Long-chain-fatty-acid--CoA ligase (EC 6.2.1.3)
Cj7	MobC-like protein
Cj7	O-acetylhomoserine sulfhydrylase (EC 2.5.1.49) @ O-succinylhomoserine sulfhydrylase (EC 2.5.1.48)
Cj7	Oxidoreductase, short-chain dehydrogenase/reductase family
Cj7	Predicted hydrolase of the alpha/beta superfamily
Cj7	putative butyryltransferase
Cj7	Putative transferase

Cj7	Putative transporter
Cj7	Pyrrolidone-carboxylate peptidase (EC 3.4.19.3)
Cj7	Transporter, LysE family
Cj7	transposase
Cj7	ulcer associated adenine specific DNA methyltransferase
Cj8	ATPase required for both assembly of type IV secretion complex and secretion of T-DNA complex, VirB11
Cj8	Coupling protein VirD4, ATPase required for T-DNA transfer
Cj8	DNA primase TraC, putative
Cj8	DNA topoisomerase III, TraE-type (EC 59912)
Cj8	FIG00471065: hypothetical protein
Cj8	helicase, Snf2 family
Cj8	IncQ plasmid conjugative transfer DNA nicking endonuclease TraR (pTi VirD2 homolog)
Cj8	IncQ plasmid conjugative transfer protein TraG
Cj8	IncQ plasmid conjugative transfer protein TraQ (RP4 TrbM homolog)
Cj8	Inner membrane protein forms channel for type IV secretion of T-DNA complex, VirB3 / ATPase required for both assembly of type IV secretion complex and secretion of T-DNA complex, VirB4
Cj8	Inner membrane protein forms channel for type IV secretion of T-DNA complex, VirB8
Cj8	Inner membrane protein of type IV secretion of T-DNA complex, TonB-like, VirB10
Cj8	Inner membrane protein of type IV secretion of T-DNA complex, VirB6
Cj8	Lipoprotein of type IV secretion complex that spans outer membrane and periplasm, VirB7
Cj8	Major pilus subunit of type IV secretion complex, VirB2
Cj8	Minor pilin of type IV secretion complex, VirB5
Cj8	Outer membrane and periplasm component of type IV secretion of T-DNA complex, has secretin-like domain, VirB9
Cj8	Replication protein
Cj8	Site-specific recombinase, resolvase family
Cj9	Conjugative transfer transglycosylase
Cj9	conserved hypothetical protein TIGR01671
Cj9	DNA primase (EC 277-)

Cj9	F-box DNA helicase 1
Cj9	FIG00470684: hypothetical protein
Cj9	FIG00471323: hypothetical protein
Cj9	FIG00471953: hypothetical protein
Cj9	IncF plasmid conjugative transfer pilus assembly protein TraB
Cj9	IncF plasmid conjugative transfer pilus assembly protein TraC
Cj9	IncF plasmid conjugative transfer pilus assembly protein TraE
Cj9	IncF plasmid conjugative transfer pilus assembly protein TraH
Cj9	IncF plasmid conjugative transfer pilus assembly protein TraK
Cj9	IncF plasmid conjugative transfer pilus assembly protein TraL
Cj9	IncF plasmid conjugative transfer pilus assembly protein TraU
Cj9	IncF plasmid conjugative transfer pilus assembly protein TraV
Cj9	IncF plasmid conjugative transfer pilus assembly protein TraW
Cj9	IncF plasmid conjugative transfer protein TraN
Cj9	IncF plasmid conjugative transfer surface exclusion protein TraT
Cj9	M protein, putative
Cj9	Phage recombination protein Bet
Cj9	Putative thioredoxin
Cj9	Thiol:disulfide involved in conjugative transfer
Cj9	Thymidine kinase (EC 27121)
Cj10	ATPase required for both assembly of type IV secretion complex and secretion of T-DNA complex, VirB4
Cj10	Domain of unknown function (DUF332) superfamily
Cj10	Erythrocyte membrane-associated antigen
Cj10	formyl transferase domain protein
Cj10	Gamma-glutamyltranspeptidase (EC 2322) @ Glutathione hydrolase (EC 341913)
Cj10	Glycosyl transferase family 8 family
Cj10	Hypothetical protein pVir0004
Cj10	Hypothetical protein pVir0007

Cj10	Hypothetical protein pVir0008
Cj10	Hypothetical protein pVir0009
Cj10	Hypothetical protein pVir0012
Cj10	Hypothetical protein pVir0015
Cj10	Hypothetical protein pVir0019/pVir0020
Cj10	Hypothetical protein pVir0029, glutamine-rich
Cj10	Hypothetical protein pVir0042
Cj10	Hypothetical protein, Lmo2313 homolog [Bacteriophage A118]
Cj10	IncF plasmid conjugative transfer protein TraG
Cj10	lipopolysaccharide core biosynthesis protein LpsA
Cj10	Mll5128 protein
Cj10	Plasmid partitioning protein ParA
Cj10	probable integral membrane protein Cj0014c
Cj10	Protein of unknown function DUF262 family
Cj10	Putative MCP-type signal transduction protein
Cj10	Putative resistance protein
Cj10	RepE replication protein, putative
Cj10	Tgh102
Cj10	thiol:disulfide interchange protein (dsbC), putative
Cj11	Adenine-specific methyltransferase (EC 21172)
Cj11	Antirestriction protein
Cj11	Bores hole in peptidoglycan layer allowing type IV secretion complex assembly to occur (VirB1)
Cj11	cell filamentation-like protein
Cj11	Conjugative transfer protein TrbB
Cj11	Conjugative transfer protein TrbD
Cj11	Conjugative transfer protein TrbE
Cj11	Conjugative transfer protein TrbF
Cj11	Conjugative transfer protein TrbI

Cj11	Conjugative transfer protein TrbL
Cj11	DNA repair protein rad2, putative
Cj11	DNA-binding protein Roi
Cj11	emm-like protein
Cj11	Endonuclease I precursor (EC 31211)
Cj11	FIG00470232: hypothetical protein
Cj11	FIG00470236: hypothetical protein
Cj11	FIG048677: hypothetical protein, phage tail fiber-like
Cj11	HNH endonuclease domain protein
Cj11	host-nuclease inhibitor protein Gam, putative
Cj11	Major capsid protein, HK97 family
Cj11	Phage baseplate assembly protein GpJ
Cj11	Phage baseplate assembly protein GpV
Cj11	Phage baseplate assembly protein GpW
Cj11	Phage DNA adenine methylase (EC 21172)
Cj11	phage head-tail adaptor, putative
Cj11	Phage head, major capsid protein E
Cj11	Phage holin/antiholin component S
Cj11	Phage integrase
Cj11	Phage major tail tube protein GpFII
Cj11	Phage portal protein
Cj11	Phage protein Mup29, H
Cj11	Phage protein Mup32, I
Cj11	phage protein, HK97 gp10 family
Cj11	phage repressor protein, putative
Cj11	Phage tail fiber protein GpH
Cj11	Phage tail formation protein GpD
Cj11	Phage tail formation protein GpI

Cj11	Phage tail length tape-measure protein GpT
Cj11	Phage tail protein E
Cj11	Phage tail protein GpU
Cj11	Phage tail protein GpX
Cj11	Phage tail sheath monomer GpFI
Cj11	Phage terminase, large subunit
Cj11	Phage terminase, small subunit
Cj11	phage uncharacterized protein
Cj11	Phage virion morphogenesis protein Mup30, F
Cj11	phage virion morphogenesis protein, putative
Cj11	Putative DNA-binding protein
Cj11	Replication initiation ATPase; bacteriophage DNA transposition B protein
Cj11	RloG protein, putative
Cj11	toxin-antitoxin protein, putative
Cj11	transfer origin protein, TraL
Cj12	CAAX amino terminal protease family protein
Cj12	cytosine specific DNA methyltransferase (BSP6IM)
Cj12	DNA methylase
Cj12	FIG00469806: hypothetical protein
Cj12	FIG00470049: hypothetical protein
Cj12	FIG00470782: hypothetical protein
Cj12	FIG00472372: hypothetical protein
Cj12	Glycerol-3-phosphate transporter
Cj12	GTP-binding protein
Cj12	LSU rRNA # 23S rRNA, large subunit ribosomal RNA - 3 prime truncation
Cj12	Outer membrane protein ImpK/VasF, OmpA/MotB domain
Cj12	oxidoreductase
Cj12	Phosphoethanolamine transferase specific for the outer Kdo residue of lipopolysaccharide

Cj12	probable acetyltransferase Cj0295
Cj12	Protein ImpG/VasA
Cj12	Putative membrane protein
Cj12	Putative oxidoreductase
Cj12	Putative transcriptional regulator
Cj12	Radical SAM domain protein
Cj12	T6SS component Hcp
Cj12	T6SS component TssB (ImpB/VipA)
Cj12	T6SS component TssC (ImpC/VipB)
Cj12	T6SS component TssK (ImpJ/VasE)
Cj12	Tgh089
Cj12	Tgh112
Cj12	transcriptional regulator, Cro/CI family
Cj12	Type I restriction-modification system, specificity subunit S (EC 3.1.21.3)
Cj12	Type II restriction adenine-specific methylase (EC 2.1.1.72)
Cj12	Uncharacterized protein similar to VCA0109
Cj12	VgrG protein
Cj13	Capsular polysaccharide biosynthesis protein
Cj13	Cell wall-associated hydrolase
Cj13	D-alanyl-D-alanine carboxypeptidase (EC 34164)
Cj13	Death on curing protein, Doc toxin
Cj13	dna methylase-type I restriction-modification system
Cj13	FIG00469675: hypothetical protein
Cj13	FIG00469749: hypothetical protein
Cj13	FIG00469921: hypothetical protein
Cj13	FIG00470126: hypothetical protein
Cj13	FIG00470444: hypothetical protein
Cj13	FIG00470625: hypothetical protein

Cj13	FIG00470651: hypothetical protein
Cj13	FIG00470885: hypothetical protein
Cj13	FIG00470993: hypothetical protein
Cj13	FIG00471428: hypothetical protein
Cj13	Hydrolase in polyol utilization gene cluster, haloacid dehalogenase-like family
Cj13	MGC82361 protein
Cj13	Nucleoside-diphosphate-sugar pyrophosphorylase involved in lipopolysaccharide biosynthesis/translation initiation factor 2B, gamma/epsilon subunits (eIF-2Bgamma/eIF-2Bepsilon)
Cj13	Phosphonate ABC transporter phosphate-binding periplasmic component (TC 3A191)
Cj13	Polysaccharide deacetylase family
Cj13	Predicted transcriptional regulator containing an HTH domain and an uncharacterized domain shared with the mammalian protein Schlafen
Cj13	Putative Dihydrolipoamide dehydrogenase (EC 1814); Mercuric ion reductase (EC 11611); PF00070 family, FAD-dependent NAD(P)-disulphide oxidoreductase
Cj13	Putative phage protein
Cj13	Tgh063
Cj13	Tgh141
Cj13	Type III restriction-modification system methylation subunit (EC 21172)
Cj13	Type III restriction-modification system restriction subunit (EC 31215)
Cj13	very hypothetical protein Cj0974
Unclassified	Adenine-specific DNA methyltransferase
Unclassified	Alcohol dehydrogenase (EC 1.1.1.1)
Unclassified	alpha-2,3-sialyltransferase
Unclassified	Anticodon nuclease
Unclassified	Bacillosamine/Legionaminic acid biosynthesis aminotransferase PglE; 4-keto-6-deoxy-N-Acetyl-D-hexosaminyl-(Lipid carrier) aminotransferase
Unclassified	bacteriophage DNA transposition protein A, putative
Unclassified	Beta-galactosidase (EC 3.2.1.23)
Unclassified	CDP-glycerol:poly(glycerophosphate) glycerophosphotransferase (EC 2.7.8.12)
Unclassified	Chitinase (EC 3.2.1.14)
Unclassified	CpsC

Unclassified	CRISPR-associated RecB family exonuclease Cas4
Unclassified	Cytidylyltransferase domain / Phosphoenolpyruvate phosphomutase (EC 5.4.2.9)
Unclassified	Dihydropyrimidine dehydrogenase [NADP+] (EC 1.3.1.2) / Pyridine nucleotide-disulphide oxidoreductase associated with reductive pyrimidine catabolism
Unclassified	DNA binding domain, excisionase family
Unclassified	DNA transposition protein
Unclassified	DNA-cytosine methyltransferase (EC 2.1.1.37)
Unclassified	Fic domain protein, MA2133 type
Unclassified	Fic family protein MloA
Unclassified	FIG00469547: hypothetical protein
Unclassified	FIG00469626: hypothetical protein
Unclassified	FIG00469776: hypothetical protein
Unclassified	FIG00469983: hypothetical protein
Unclassified	FIG00470037: hypothetical protein
Unclassified	FIG00470129: hypothetical protein
Unclassified	FIG00470265: hypothetical protein
Unclassified	FIG00470419: hypothetical protein
Unclassified	FIG00470447: hypothetical protein
Unclassified	FIG00471393: hypothetical protein
Unclassified	FIG00471437: hypothetical protein
Unclassified	FIG00471554: hypothetical protein
Unclassified	FIG00471635: hypothetical protein
Unclassified	FIG00471674: hypothetical protein
Unclassified	FIG00471942: hypothetical protein
Unclassified	FIG00711288: hypothetical protein
Unclassified	FIG01210424: hypothetical protein
Unclassified	Formyl transferase domain protein
Unclassified	Glycerate kinase (EC 2.7.1.31)

Unclassified	Glycerol-3-phosphate cytidylyltransferase (EC 2.7.7.39)
Unclassified	Glycosyl transferase, family 2
Unclassified	Group-specific protein
Unclassified	Hypothetical protein Cj0566
Unclassified	Hypothetical protein Cj0569
Unclassified	Hypothetical protein pVir0016
Unclassified	IncF plasmid conjugative transfer protein TraD
Unclassified	Indolepyruvate ferredoxin oxidoreductase, alpha and beta subunits
Unclassified	Inner membrane protein YihY, formerly thought to be RNase BN
Unclassified	LSU rRNA # 23S rRNA, large subunit ribosomal RNA - 5 prime truncation
Unclassified	LSU rRNA # 23S rRNA, large subunit ribosomal RNA - 5 prime truncation - 3 prime truncation
Unclassified	MCP-domain signal transduction protein
Unclassified	mRNA interferase YafQ
Unclassified	N-Acetyltransferase PseH involved in the biosynthesis of pseudaminic acid
Unclassified	Nucleoside-diphosphate-sugar pyrophosphorylase involved in lipopolysaccharide biosynthesis/translation initiation factor 2B, gamma/epsilon subunits (eIF-2Bgamma/eIF-2Bepsilon) / Nucleoside-diphosphate-sugar pyrophosphorylase involved in lipopolysaccharide biosynthesis/translation initiation factor 2B, gamma/epsilon subunits (eIF-2Bgamma/eIF-2Bepsilon)
Unclassified	Nucleotide sugar dehydratase, putative
Unclassified	Pentapeptide repeat family protein
Unclassified	Phage antirepressor protein
Unclassified	Phage major capsid protein
Unclassified	phage major tail tube protein, putative
Unclassified	Phage protein
Unclassified	phage putative head morphogenesis protein, SPP1 gp7 family
Unclassified	Phage tail formation protein D
Unclassified	Phage tail length tape-measure protein T
Unclassified	Phosphate transport system permease protein PstA (TC 3.A.1.7.1)
Unclassified	Phosphomevalonate kinase (EC 2.7.4.2)
Unclassified	Phosphonopyruvate decarboxylase (EC 4.1.1.82)

Unclassified	Possible lipoprotein
Unclassified	Possible outer membrane protein
Unclassified	predicted secreted protein
Unclassified	prophage ps3 protein 01
Unclassified	putative antirepressor
Unclassified	putative DNA helicase
Unclassified	putative serine/threonine protein kinase
Unclassified	putative TYPE II DNA MODIFICATION ENZYME (METHYLTRANSFERASE)
Unclassified	Putrescine transport ATP-binding protein PotA (TC 3.A.1.11.1)
Unclassified	Replicative DNA helicase (EC 3.6.1.-)
Unclassified	RloF
Unclassified	Serine/threonine protein kinase PrkC, regulator of stationary phase
Unclassified	Sugar phosphate permease of the Major Facilitator Superfamily
Unclassified	terminase B protein, putative
Unclassified	Tol-Pal system-associated acyl-CoA thioesterase
Unclassified	tRNA-Pseudo-TCT
Unclassified	Uncharacterized protein ImpA

Supplemental Table 5. Protein encoding genes belonging to *Campylobacter coli* (n=13 isolates) variable genomes.

**Cluster**	**Protein-encoding gene**
Cc1	3-oxoacyl-[acyl-carrier protein] reductase (EC 1.1.1.100)
Cc1	C4-dicarboxylate anaerobic carrier, putative
Cc1	Capsular polysaccharide biosynthesis heptosyltransferase HddD
Cc1	CDP-4-dehydro-6-deoxy-D-glucose 3-dehydratase (EC 4.2.1.-)
Cc1	CDP-abequose synthase
Cc1	conserved hypothetical protein TIGR01671
Cc1	D-glycero-alpha-D-manno-heptose 1-phosphate guanylyltransferase (EC 2.7.7.71)
Cc1	D-glycero-alpha-D-manno-heptose 7-phosphate kinase (EC 2.7.1.168)
Cc1	dTDP-4-dehydrorhamnose 3,5-epimerase (EC 5.1.3.13)
Cc1	Endonuclease I precursor (EC 3.1.21.1)
Cc1	Enterochelin uptake permease CeuB
Cc1	FIG00469658: hypothetical protein
Cc1	FIG00469879: hypothetical protein
Cc1	GDP-L-fucose synthetase (EC 1.1.1.271)
Cc1	GDP-mannose 4,6-dehydratase (EC 4.2.1.47)
Cc1	Glucose-1-phosphate cytidylyltransferase (EC 2.7.7.33)
Cc1	Glycerate kinase (EC 2.7.1.31)
Cc1	host-nuclease inhibitor protein Gam, putative
Cc1	membrane protein , putative
Cc1	membrane protein, putative
Cc1	methyl-accepting chemotaxis protein, putative
Cc1	oligopeptide transporter
Cc1	oxidoreductase of aldo/keto reductase family, subgroup 1
Cc1	Phage baseplate assembly protein GpJ
Cc1	Phage baseplate assembly protein GpV

Cc1	Phage baseplate assembly protein GpW
Cc1	Phage DNA adenine methylase (EC 2.1.1.72)
Cc1	Phage head, major capsid protein E
Cc1	Phage holin/antiholin component S
Cc1	Phage major tail tube protein GpFII
Cc1	Phage protein Mup29, H
Cc1	Phage protein Mup32, I
Cc1	phage repressor protein, putative
Cc1	Phage tail fiber protein GpH
Cc1	Phage tail formation protein GpD
Cc1	Phage tail formation protein GpI
Cc1	Phage tail length tape-measure protein GpT
Cc1	Phage tail protein E
Cc1	Phage tail protein GpU
Cc1	Phage tail protein GpX
Cc1	Phage tail sheath monomer GpFI
Cc1	phage uncharacterized protein
Cc1	Phage virion morphogenesis protein Mup30, F
Cc1	phage virion morphogenesis protein, putative
Cc1	Putative DNA-binding protein
Cc1	Replication initiation ATPase; bacteriophage DNA transposition B protein
Cc1	Sugar phosphate permease of the Major Facilitator Superfamily
Cc1	UDP-glucose 6-dehydrogenase (EC 1.1.1.22)
Cc1	UDP-glucuronate decarboxylase (EC 4.1.1.35)
Cc2	2-dehydro-3-deoxyglucarate aldolase (EC 4.1.2.20)
Cc2	3-oxoacyl-[acyl-carrier-protein] synthase, KASIII (EC 2.3.1.41)
Cc2	ATPase required for both assembly of type IV secretion complex and secretion of T-DNA complex, VirB11
Cc2	cag pathogenicity island protein (cag12)

Cc2	capsular polysaccharide biosynthesis protein, putative
Cc2	conserved hypothetical protein 22
Cc2	Coupling protein VirD4, ATPase required for T-DNA transfer
Cc2	Cytidine diphosphoramidate kinase Cj1415
Cc2	Cytolethal distending toxin subunit C
Cc2	DNA primase (EC 2.7.7.-)
Cc2	DNA primase TraC, putative
Cc2	DNA topoisomerase III, TraE-type (EC 5.99.1.2)
Cc2	dTDP-3-amino-3,6-dideoxy-alpha-D-galactopyranose 3-N-acetyltransferase (EC 2.3.1.197)
Cc2	dTDP-glucose 4,6-dehydratase (EC 4.2.1.46)
Cc2	Fic family protein MloA
Cc2	FIG00469638: hypothetical protein
Cc2	FIG00469761: hypothetical protein
Cc2	FIG00470572: hypothetical protein
Cc2	FIG00471048: hypothetical protein
Cc2	FIG00471065: hypothetical protein
Cc2	FIG00471323: hypothetical protein
Cc2	FIG00471788: hypothetical protein
Cc2	formyl transferase domain protein
Cc2	Galactoside O-acetyltransferase(EC:2.3.1.79)
Cc2	gamma-Glutamyl-CDP-amidate hydrolase involved in O-methyl phosphoramidate capsule modification
Cc2	Glucose-1-phosphate thymidylyltransferase (EC 2.7.7.24)
Cc2	Glycosyl transferase, group 2 family protein
Cc2	helicase, Snf2 family
Cc2	hydrolase, haloacid dehalogenase-like family
Cc2	IncQ plasmid conjugative transfer DNA nicking endonuclease TraR (pTi VirD2 homolog)
Cc2	IncQ plasmid conjugative transfer protein TraG
Cc2	IncQ plasmid conjugative transfer protein TraQ (RP4 TrbM homolog)

Cc2	Inner membrane protein forms channel for type IV secretion of T-DNA complex, VirB3 / ATPase required for both assembly of type IV secretion complex and secretion of T-DNA complex, VirB4
Cc2	Inner membrane protein forms channel for type IV secretion of T-DNA complex, VirB8
Cc2	Inner membrane protein of type IV secretion of T-DNA complex, TonB-like, VirB10
Cc2	Inner membrane protein of type IV secretion of T-DNA complex, VirB6
Cc2	Iron compound ABC uptake transporter permease protein
Cc2	L-glutamine kinase involved in O-methyl phosphoramidate capsule modification
Cc2	Lipoprotein of type IV secretion complex that spans outer membrane and periplasm, VirB7
Cc2	Major pilus subunit of type IV secretion complex, VirB2
Cc2	Methyltransferase (EC 2.1.1.-), possibly involved in O-methyl phosphoramidate capsule modification
Cc2	Minor pilin of type IV secretion complex, VirB5
Cc2	NAD-dependent epimerase/dehydratase
Cc2	Nucleoside-diphosphate-sugar pyrophosphorylase involved in lipopolysaccharide biosynthesis/translation initiation factor 2B, gamma/epsilon subunits (eIF-2Bgamma/eIF-2Bepsilon) / Nucleoside-diphosphate-sugar pyrophosphorylase involved in lipopolysaccharide biosynthesis/translation initiation factor 2B, gamma/epsilon subunits (eIF-2Bgamma/eIF-2Bepsilon)
Cc2	Outer membrane and periplasm component of type IV secretion of T-DNA complex, has secretin-like domain, VirB9
Cc2	Phage Rha protein
Cc2	Phosphoglutamine cytidylyltransferase (EC 2.7.7.-) in O-methyl phosphoramidate capsule modification
Cc2	Possible sugar transferase
Cc2	Replication protein
Cc2	short chain dehydrogenase
Cc2	Site-specific recombinase, resolvase family
Cc2	Type I restriction-modification system, restriction subunit R (EC 3.1.21.3)
Cc2	Type I restriction-modification system, specificity subunit S

Supplemental Table 6. Antimicrobial resistance gene (ARG) profiles.

**ARG profile**	**Total number of isolates with the ARG profile**	***C. coli* isolates with the ARG profile**	***C. jejuni* isolates with the ARG profile**
50S_L22_A103V / *bla*OXA-603	3	0	3
50S_L22_A103V / *gyrA*_T86I / *tetO*	1	0	1
aadE-Cc / *bla*OXA-193	7	7	0
aph(3')-IIIa / *bla*OXA-193 / *gyrA*_T86I / *tetO*	2	0	2
aph(3')-IIIa / *bla*OXA-193 / *tetO*	8	0	8
aph(3')-IIIa / *tetO*	1	0	1
*bla*OXA-61	1	0	1
*bla*OXA-193	15	1	14
*bla*OXA-193 / *gyrA*_T86I / *tetO*	5	5	0
*bla*OXA-193 / *tetO*	8	0	8
*bla*OXA-449 / *tetO*	3	0	3
*bla*OXA-461 / *tetO*	5	0	5
*rpsL*_K88R	1	0	1
tetO	5	0	5

Supplemental Table 7. Antimicrobial resistance gene (ARG) profiles per sequence type (ST) and clonal complex (CC).

Species	CC	ST	ARG profile	Number of isolates
C. coli	828	829	*aadE-Cc* / *bla*OXA-193	7
	828	829	*bla*OXA-193	1
	828	1068	*bla*OXA-193 / *gyrA*_T86I / *tetO*	5
C. jejuni	21	8	*bla*OXA-193 / *tetO*	5
	21	21	*tetO*	1
	21	21	*aph(3')-IIIa* / *bla*OXA-193 / *gyrA*_T86I / *tetO*	1
	21	806	*aph(3')-IIIa* / *bla*OXA-193 / *gyrA*_T86I / *tetO*	1
	21	806	*aph(3')-IIIa* / *bla*OXA-193 / *tetO*	3
	21	922	*aph(3')-IIIa* / *bla*OXA-193 / *tetO*	5
	21	922	*bla*OXA-193	5
	21	929	*bla*OXA-461 / *tetO*	3
	21	982	*bla*OXA-193 / *tetO*	2
	42	42	None	2
	42	459	*aph(3')-IIIa* / *tetO*	1
	42	459	tetO	3
	42	604	*rpsL*_K88R	1
	42	11712	*tetO*	1
	42	11771	*tetO*	1
	45	45	*bla*OXA-449 / *tetO*	3
	48	48	*bla*OXA-61	1
	52	52	*50S_L22_A103V* / *bla*OXA-603	3
	61	61	*bla*OXA-193	7
	61	1244	*bla*OXA-193 / *tetO*	1
	257	5261	*bla*OXA-461 / *tetO*	2
	283	267	*bla*OXA-193	1
	403	403	None	2
	464	464	*50S_L22_A103V* / *gyrA*_T86I / *tetO*	1

Supplemental Table 8. Co-occurrence profile of *Campylobacter coli* variable genomes (n=13 isolates)

Cluster	Protein-encoding gene
	outer membrane and periplasm component of type IV secretion of T-DNA complex, has secretin-like domain, VirB9
	Tetracycline resistance, ribosomal protection type => Tet(O)
	capsular polysaccharide biosynthesis protein, putative
	gamma-Glutamyl-CDP-amidate hydrolase involved in O-methyl phosphoramidate capsule modification
	L-glutamine kinase involved in O-methyl phosphoramidate capsule modification
	Methyltransferase (EC 2.1.1.-), possibly involved in O-methyl phosphoramidate capsule modification
	Phosphoglutamine cytidylyltransferase (EC 2.7.7.-) in O-methyl phosphoramidate capsule modification
	ATPase required for both assembly of type IV secretion complex and secretion of T-DNA complex, VirB11
	Coupling protein VirD4, ATPase required for T-DNA transfer
	DNA primase (EC 2.7.7.-)
	DNA primase TraC, putative
	DNA topoisomerase III, TraE-type (EC 5.99.1.2)
	IncQ plasmid conjugative transfer DNA nicking endonuclease TraR (pTi VirD2 homolog)
	IncQ plasmid conjugative transfer protein TraG
	IncQ plasmid conjugative transfer protein TraQ (RP4 TrbM homolog)
	Inner membrane protein forms channel for type IV secretion of T-DNA complex, VirB3 / ATPase required for both assembly of type IV secretion complex and secretion of T-DNA complex, VirB4
	Inner membrane protein forms channel for type IV secretion of T-DNA complex, VirB8
	Inner membrane protein of type IV secretion of T-DNA complex, TonB-like, VirB10
	Inner membrane protein of type IV secretion of T-DNA complex, VirB6
	Lipoprotein of type IV secretion complex that spans outer membrane and periplasm, VirB7
	Major pilus subunit of type IV secretion complex, VirB2
	Minor pilin of type IV secretion complex, VirB5
Conjugation / type 4 secretion system cluster	Outer membrane and periplasm component of type IV secretion of T-DNA complex, has secretin-like domain, VirB9
	Phage Rha protein
	cag pathogenicity island protein (cag12)
	Cytidine diphosphoramidate kinase Cj1415

	Type I restriction-modification system, restriction subunit R (EC 3.1.21.3)
	3-oxoacyl-[acyl-carrier-protein] synthase, KASIII (EC 2.3.1.41)
	Fic family protein MloA
	Glucose-1-phosphate thymidylyltransferase (EC 2.7.7.24)
	dTDP-glucose 4,6-dehydratase (EC 4.2.1.46)
	2-dehydro-3-deoxyglucarate aldolase (EC 4.1.2.20)
	Cytolethal distending toxin subunit C
	dTDP-3-amino-3,6-dideoxy-alpha-D-galactopyranose 3-N-acetyltransferase (EC 2.3.1.197)
	formyl transferase domain protein
	Galactoside O-acetyltransferase(EC:2.3.1.79)
	Glycosyl transferase, group 2 family protein
	helicase, Snf2 family
	hydrolase, haloacid dehalogenase-like family
	Iron compound ABC uptake transporter permease protein
	NAD-dependent epimerase/dehydratase
	Nucleoside-diphosphate-sugar pyrophosphorylase involved in lipopolysaccharide biosynthesis/translation initiation factor 2B, gamma/epsilon subunits (eIF-2Bgamma/eIF-2Bepsilon) / Nucleoside-diphosphate-sugar pyrophosphorylase involved in lipopolysaccharide biosynthesis/translation initiation factor 2B, gamma/epsilon subunits (eIF-2Bgamma/eIF-2Bepsilon)
	Possible sugar transferase
	Replication protein
	short chain dehydrogenase
	Site-specific recombinase, resolvase family
	Type I restriction-modification system, specificity subunit S
	Enterochelin uptake permease CeuB
	3-oxoacyl-[acyl-carrier protein] reductase (EC 1.1.1.100)
	GDP-L-fucose synthetase (EC 1.1.1.271)
	host-nuclease inhibitor protein Gam, putative
	Glycerate kinase (EC 2.7.1.31)
	GDP-mannose 4,6-dehydratase (EC 4.2.1.47)
Prophage cluster	dTDP-4-dehydrorhamnose 3,5-epimerase (EC 5.1.3.13)

	Glucose-1-phosphate cytidylyltransferase (EC 2.7.7.33)
	UDP-glucose 6-dehydrogenase (EC 1.1.1.22)
	C4-dicarboxylate anaerobic carrier, putative
	CDP-4-dehydro-6-deoxy-D-glucose 3-dehydratase (EC 4.2.1.-)
	CDP-abequose synthase
	conserved hypothetical protein TIGR01671
	Endonuclease I precursor (EC 3.1.21.1)
	membrane protein , putative
	membrane protein, putative
	methyl-accepting chemotaxis protein, putative
	oligopeptide transporter
	oxidoreductase of aldo/keto reductase family, subgroup 1
	Sugar phosphate permease of the Major Facilitator Superfamily
	UDP-glucuronate decarboxylase (EC 4.1.1.35)
	Putative DNA-binding protein
	D-glycero-alpha-D-manno-heptose 7-phosphate kinase (EC 2.7.1.168)
	D-glycero-alpha-D-manno-heptose 1-phosphate guanylyltransferase (EC 2.7.7.71)
	Capsular polysaccharide biosynthesis heptosyltransferase HddD
	Phage baseplate assembly protein GpJ
	Phage baseplate assembly protein GpV
	Phage baseplate assembly protein GpW
	Phage DNA adenine methylase (EC 2.1.1.72)
	Phage head, major capsid protein E
	Phage holin/antiholin component S
	Phage major tail tube protein GpFII
	Phage protein Mup29, H
	Phage protein Mup32, I
	phage repressor protein, putative
	Phage tail fiber protein GpH
	Phage tail formation protein GpD

	Phage tail formation protein GpI
	Phage tail length tape-measure protein GpT
	Phage tail protein E
	Phage tail protein GpU
	Phage tail protein GpX
	Phage tail sheath monomer GpFI
	phage uncharacterized protein
	Phage virion morphogenesis protein Mup30, F
	phage virion morphogenesis protein, putative
	Replication initiation ATPase; bacteriophage DNA transposition B protein
Arsenical resistance cluster	aminoglycoside 6-adenylyltransferase
	Arsenate reductase (EC 1.20.4.4) thioredoxin-coupled, LMWP family
	Arsenical resistance operon repressor
	Arsenical-resistance protein ACR3
	Dienelactone hydrolase and related enzymes
	RecD-like DNA helicase YrrC
	Uncharacterized membrane protein, YraQ family
	Uncharacterized NAD(P)H oxidoreductase, YdeQ/YrkL/YwrO family

Supplemental Table 9. Antibiotic resistance genes located on plasmids identified by MOB-Suite.

**species**	**NCBI_id**	**contig_id**	**p_cluster**	**p_neighbor**	**ARG_gene**	**ARG_class**
Cj	113002	Contig_30_81.5577	AC321	CP045046	*tet(O)*	tetracycline
Cj	113002	Contig_30_81.5577	AC321	CP045046	*aph(3')-IIIa*	aminoglycoside
Cc	112154	Contig_18_281.007	AC320	CP017418	*tet(O)*	tetracycline
Cc	112156	Contig_12_290.262	AC320	CP017418	*tet(O)*	tetracycline
Cj	112944	Contig_2_155.04	AC320	AY394561	*tet(O)*	tetracycline
Cc	112141	Contig_16_86.1009	AC320	CP017418	*tet(O)*	tetracycline
Cc	112142	Contig_27_122.643	AC320	CP017418	*tet(O)*	tetracycline
Cj	112992	Contig_34_165.248	AC320	KJ646012	*tet(O)*	tetracycline
Cj	112992	Contig_38_168.852	AC320	KJ646012	*aph(3')-IIIa*	aminoglycoside
Cj	112936	Contig_11_151.827	AC320	CP013035	*tet(O)*	tetracycline
Cj	112936	Contig_11_151.827	AC320	CP013035	*aph(3')-IIIa*	aminoglycoside
Cj	112949	Contig_13_136.708	AC321	CP045046	*tet(O)*	tetracycline
Cj	112949	Contig_13_136.708	AC321	CP045046	*aph(3')-IIIa*	aminoglycoside
Cj	113016	Contig_33_191.185	AC320	KJ646012	*tet(O)*	tetracycline
Cj	113016	Contig_33_191.185	AC320	KJ646012	*aph(3')-IIIa*	aminoglycoside
Cj	112958	Contig_8_186.212	AC320	CP007752	*tet(O)*	tetracycline
Cj	112958	Contig_8_186.212	AC320	CP007752	*aph(3')-IIIa*	aminoglycoside
Cj	112930	Contig_73_233.894	AC320	CP013035	*tet(O)*	tetracycline
Cj	112930	Contig_73_233.894	AC320	CP013035	*aph(3')-IIIa*	aminoglycoside
Cj	112961	Contig_33_281.273	AC321	CP045046	*tet(O)*	tetracycline
Cj	112961	Contig_33_281.273	AC321	CP045046	*aph(3')-IIIa*	aminoglycoside
Cj	112924	Contig_45_189.151	AC320	CP017416	*tet(O)*	tetracycline
Cj	112924	Contig_45_189.151	AC320	CP017416	*aph(3')-IIIa*	aminoglycoside
Cj	112167	Contig_7_131.203	AE190	CP044174	*tet(O)*	tetracycline
Cj	112981	Contig_16_105.628	AC320	AY394561	*tet(O)*	tetracycline
Cj	112927	Contig_20_165.944	AE015	CP007184	*tet(O)*	tetracycline
Cj	112927	Contig_20_165.944	AE015	CP007184	*aph(3')-IIIa*	aminoglycoside

Cj	113230	Contig_7_211.859	novel_a7ef41ed15871c6b12255249eb28cb44	MK251542	*tet(O)*	tetracycline
Cc	112127	Contig_1_244.183_Circ	AC320	CP017418	*tet(O)*	tetracycline
Cj	113015	Contig_21_539.177	AC321	CP045046	*tet(O)*	tetracycline
Cj	113015	Contig_21_539.177	AC321	CP045046	*aph(3')-IIIa*	aminoglycoside

"p_cluster": MOB-Suite plasmid cluster; "p_neighbor": accession number of the Mash distance nearest neighbor of the plasmid. *Cj* and *Cc*: *Campylobacter jejuni* and *coli*, respectively.

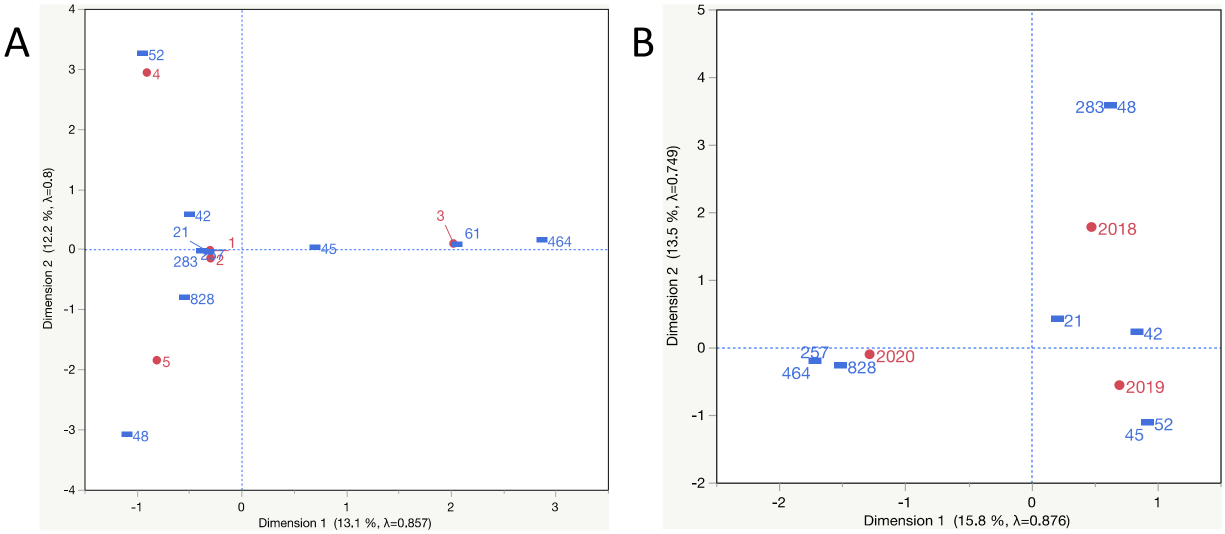

Supplemental Figure 1. Multi-correspondence analysis of clonal complexes (CC; blue label) by farm clusters (1-5; red label in Figure 1A) and year of collection (2018-2020; red label in Figure 1B). The distance between the CC indicates the likeliness that these CC can be found within the same farm cluster or year of collection. The distance between the farm clusters or the year of collection indicates the CC profile similarity between the farm clusters or years of collection.

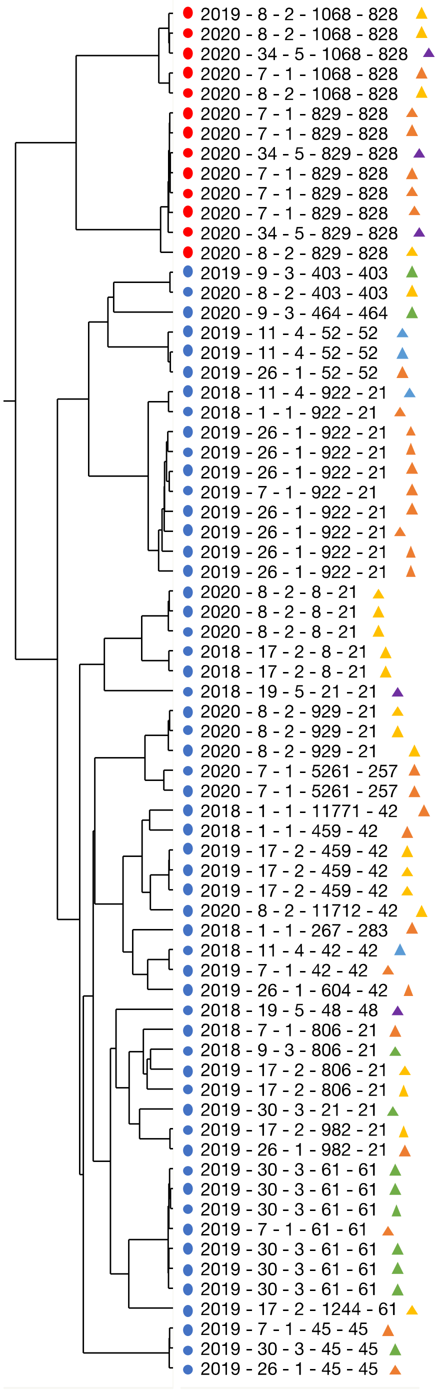

Supplemental Figure 2. Core-genome multi locus sequence typing (cgMLST) profile among the 69 *Campylobacter* isolated from dairy manure in North East Ohio. The tree was generated based on the cgMLST data. Each row indicates a specific *Campylobacter* isolate (year of collection - farm ID - farm cluster - sequence type - clonal complex). Blue and red circles indicate *Campylobacter jejuni* and *coli*, respectively. Colored triangles are associated with the farm cluster number; Orange, yellow, green, blue and purple triangles indicate farm cluster 1 to 5, respectively.

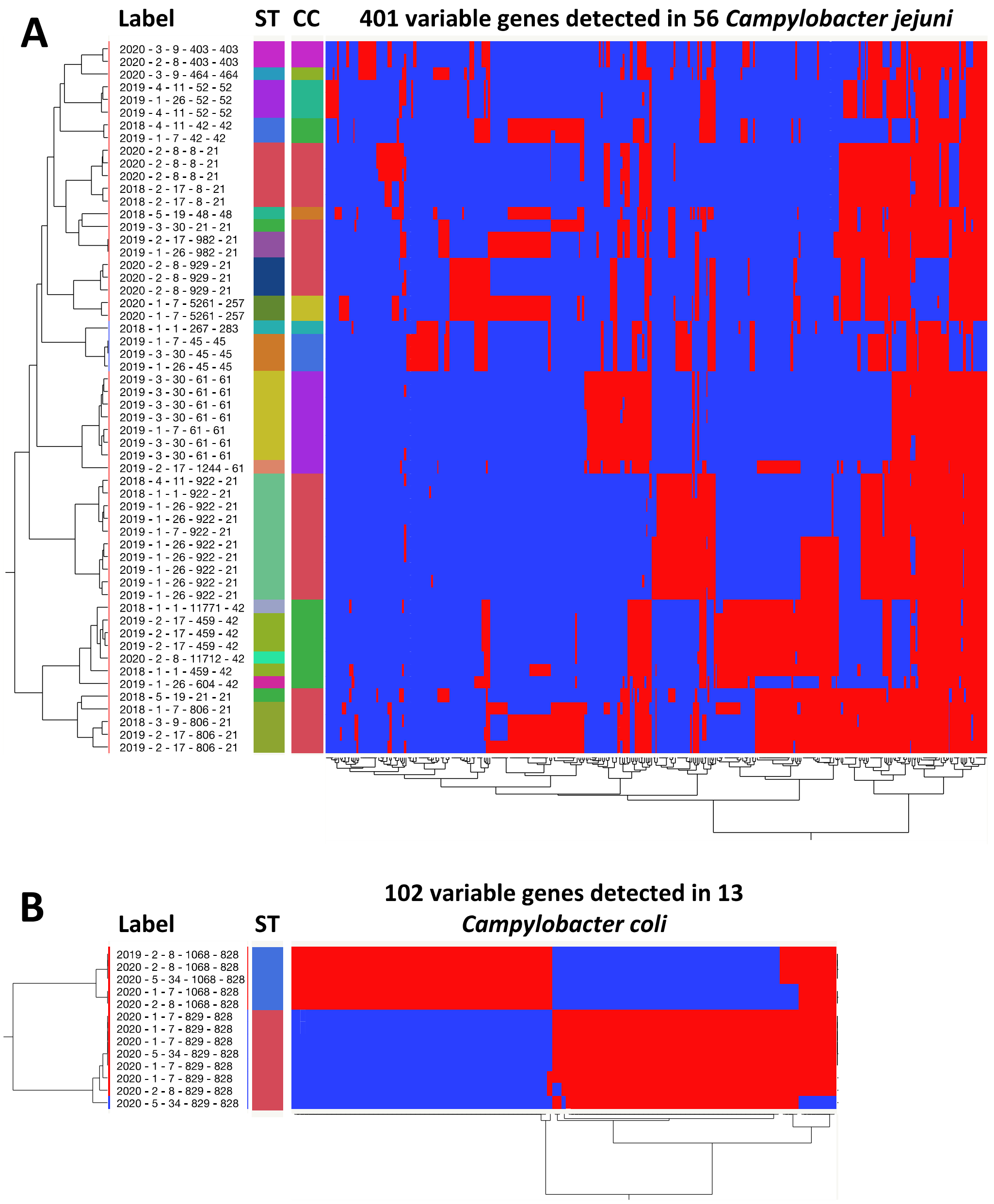

Supplemental Figure 3. Variable-genome profile among the 69 *Campylobacter* isolated from dairy manure in North East Ohio. A) Variable-genome profile of the 56 C. *jejuni* (n=401variable genes) and B) variable-genome profile of the 13 *C. coli* isolates (n= 102 variable genes). The figures were generated based on the presence/absence of annotated genes (red and blue cells, respectively) using a Ward's hierarchical clustering analysis in JMP Pro 16 (SAS, Cary, NC). Each row indicates a specific *Campylobacter* isolate (year of collection - farm cluster - farm ID - sequence type [ST]- clonal complex [CC]). Colored cells in the first column are associated with the ST number, while colored cells in the second column are associated with the CC number. No CC column was added to the *C. coli* figure given all isolates belongs to CC-828. Tree on the Y axis indicates the distribution of the *Campylobacter* isolates based on their variable genome profile, while the Tree on the X axis indicates the distribution of the protein-encoding genes based on their presence/absence on the *Campylobacter* isolates.

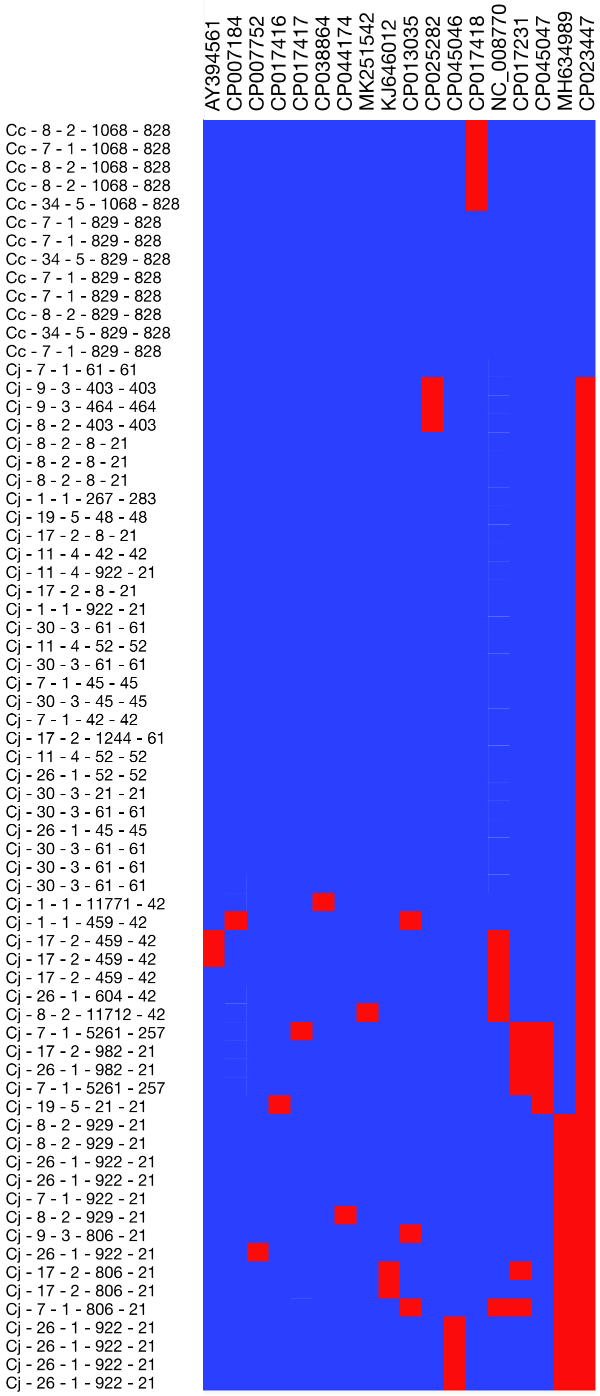

Supplemental Figure 4. Plasmid composition in the 69 *Campylobacter* isolated from dairy manure. Red and blue cells represent presence and absence of the designated plasmid (column), respectively. Each row indicates a specific *Campylobacter* isolate (species - farm ID - farm cluster - sequence type - clonal complex). *Cj* and *Cc*: *Campylobacter jejuni* and *coli*, respectively. Only *C. coli* ST-1069 CC-828 isolates (n=5) possessed plasmid accession number CP017418 (also called pMTVDSCj13-1). Interestingly, no plasmid was detected in *C. coli* ST-829 CC-828 isolates (n=8). All C. *jejuni* isolates (n=56) possessed the plasmid accession number CP023447 (also called pJc100). Most *C. jejuni* CC-21 (n=15/26) including ST-929 (n=3/3), ST-

922 (n=8/10) and ST-806 (n=4/4) possessed the plasmid accession number MH634989 (also called pCCDM18S1). *C. jejuni* CC-21 ST-922 (n=4/10) also possessed the plasmid accession number CP045046 (also called p20827S). Most *C. jejuni* ST-459 CC-42 (n=3/4) possessed the plasmid accession number NC_008770 (also called pVir). All *C. jejuni* ST-5261 CC-257 (n=2) and ST-982 CC-21 (n=2) possessed the plasmid accession number CP045047 (also called p20827S) and CP017231 (also called pFORC46.2). All *C. jejuni* ST-403 CC-403 (n=2) and ST-464 CC-464 (n=1) possessed the plasmid accession number CP025282 (also called pCOS503).
